# Methionine adenosyltransferase2A inhibition restores metabolism to improve regenerative capacity and strength of aged skeletal muscle

**DOI:** 10.1038/s41467-023-36483-3

**Published:** 2023-02-16

**Authors:** Nika Rajabian, Izuagie Ikhapoh, Shahryar Shahini, Debanik Choudhury, Ramkumar Thiyagarajan, Aref Shahini, Joseph Kulczyk, Kendall Breed, Shilpashree Saha, Mohamed Alaa Mohamed, Susan B. Udin, Aimee Stablewski, Kenneth Seldeen, Bruce R. Troen, Kirkwood Personius, Stelios T. Andreadis

**Affiliations:** 1 Department of Chemical and Biological Engineering, University at Buffalo, The State University of New York, Amherst, NY USA; 2 Division of Geriatrics and Palliative Medicine, Jacobs School of Medicine and Biomedical Sciences, University at Buffalo and Research Service, Veterans Affairs Western New York Healthcare System, Buffalo, NY USA; 3 Department of Biomedical Engineering, University at Buffalo, Amherst, NY USA; 4 Department of Physiology and Biophysics, School of Medicine and Biomedical Sciences, University at Buffalo, Buffalo, NY USA; 5 Gene Targeting and Transgenic Shared Resource, Roswell Park Comprehensive Cancer Institute, Buffalo, NY USA; 6 Department of Rehabilitation Science, School of Public Health and Health Professions, University at Buffalo, Buffalo, NY USA; 7 Center of Excellence in Bioinformatics and Life Sciences, Buffalo, NY USA; 8 Cell, Gene and Tissue Engineering (CGTE) Center, School of Engineering and Applied Sciences, University at Buffalo, Amherst, NY USA

**Keywords:** Ageing, Molecular medicine, Muscle stem cells, Regeneration

## Abstract

We investigate the age-related metabolic changes that occur in aged and rejuvenated myoblasts using in vitro and in vivo models of aging. Metabolic and signaling experiments reveal that human senescent myoblasts and myoblasts from a mouse model of premature aging suffer from impaired glycolysis, insulin resistance, and generate Adenosine triphosphate by catabolizing methionine via a methionine adenosyl-transferase 2A-dependant mechanism, producing significant levels of ammonium that may further contribute to cellular senescence. Expression of the pluripotency factor NANOG downregulates methionine adenosyltransferase 2 A, decreases ammonium, restores insulin sensitivity, increases glucose uptake, and enhances muscle regeneration post-injury. Similarly, selective inhibition of methionine adenosyltransferase 2 A activates Akt2 signaling, repairs pyruvate kinase, restores glycolysis, and enhances regeneration, which leads to significant enhancement of muscle strength in a mouse model of premature aging. Collectively, our investigation indicates that inhibiting methionine metabolism may restore age-associated impairments with significant gain in muscle function.

## Abstract (web summary)

Aged myoblasts suffer from impaired glycolysis and insulin resistance, but increase methionine catabolism, possibly to meet energetic demands. Here, authors show that inhibiting methionine metabolism via NANOG reprogramming or MAT2A inhibition restores the function and regeneration capacity of aged muscle.

## Introduction

Skeletal Muscle (SkM) constitutes approximately 40% of the total body mass and carries important physiological functions such as producing skeletal movements, maintaining body posture, and regulating body temperature. Age-related loss in muscle mass and function, is a major challenge facing older adults and correlates with disabilities, loss of metabolic function, impaired bioenergetics^1^, morbidity^1^, and mortality^2^. Muscle regeneration relies on the activation of myogenic progenitors but the number of progenitor cells and their regenerative capacity decline with aging and cellular senescence^3^.

Hutchinson-Gilford Progeria Syndrome (HGPS), also known as progeria, is the result of a mutation in the nuclear filament LMNA gene, leading to truncation of the Lamin A protein and, therefore, aberrant accumulation of progerin that destabilizes nuclear membranes^4^, ultimately causing premature aging and death^5–8^. Typically, a 7-year-old progeric child may display the cellular and physical traits of a 70-year-old^7^ and may die from age-related diseases such as heart attack or stroke^9,10^; while most of the surviving children continue to face major medical challenges that lead to poor quality of life^11^, including muscle loss^12^. Interestingly, the importance of nuclear lamins has also been documented in normal aging as evidenced by the accumulation of permanently farnesylated pre-lamin A in aging tissues in vivo causing DNA damage and mitotic defects^13^. Accumulating evidence suggests that progerin levels may increase with age and contribute to cellular defects that are associated with normal aging including atherosclerotic plaques and vascular stiffening^9,14,15^. Therefore, progeric mice and cellular models have been used to study age-related disorders including muscle loss^16^.

A recent study demonstrated that a methionine-restricted (MR)^17^ diet improved mitochondrial function and upregulated autophagy-related genes, resulting in a 45% extension of rodent lifespan^18^. Other studies also demonstrated that MR regulates energy expenditure in the aged musculoskeletal system^19^, activated insulin signaling that improved type II diabetes^20,21^, and decreased lipid peroxidation that reduced hyperlipidemia^21,22^. Clearly, decreasing dietary methionine has beneficial physiologic effects. However, a pathway connecting methionine to these pathologies is yet to be elucidated. Methionine breakdown begins with MAT2A, which catalyzes the production of *S*-adenosyl methionine (SAMe) from methionine^23^ and is associated with various diseases including familial thoracic aortic aneurysm^24^ and aortic dissection both of which affect the smooth muscle layer of vasculature^24^.

Previously, our laboratory reported similar pathologies affecting cells from progeria patients and cells undergone replicative senescence^25,26^. Further, we demonstrated that with ectopic expression of the pluripotency factor, NANOG could effectively reverse aging hallmarks and reestablish young attributes in older cells to restore their myogenic differentiation capacity^26^, decrease senescence-associated beta-galactosidase (SA-βgal), restore mitochondrial function, and repair DNA damage^26^. NANOG also restored the ability of SM to differentiate into healthy skeletal myotubes^27^ and ameliorated the hallmarks of cellular senescence including genomic instability, loss of proteostasis, and mitochondrial dysfunction in human skeletal myoblasts and restored the number of myogenic progenitors in a mouse model of premature aging^26^. However, the mechanism through which NANOG imparts its rejuvenating effects is not known. Using NANOG as an investigative tool, we examined whether metabolic impairments due to senescence or premature aging could be reversed, restoring muscle function. We discovered that senescent myoblasts used methionine to meet their metabolic demands and that increased use of methionine contributed to the loss of skeletal muscle function. Conversely, inhibition of MAT2A catabolism by NANOG expression or chemical inhibition restored glucose-based bioenergetics and the force-generating capacity of aged skeletal muscle in a mouse model of premature aging.

## Results

### Impaired glycolytic capacity is restored in rejuvenated human myoblasts in vitro

Recently, we assessed well-accepted hallmarks of aging including expression of SA-β-gal, cellular morphology, DNA damage, histone modifications, and their ability to form myotubes as cells were cultured for different times (passages)^26,28^. Human myoblasts that were cultured for less than seven passages showed low levels of SA-β-gal expression (15 ± 3.3%), short doubling time (48 h), small cell size (diameter = 18.65 ± 0.92 µm), and formed robust and contractile myotubes that exhibited high regeneration capacity upon injury. In contrast, myoblasts that were cultured for more than ten passages exhibited hallmarks of senescence including a high percentage of SA-β-gal+ cells (72 ± 8.5%), much longer doubling time (98 ± 3.9 h), and significantly increased cell size (diameter = 36.81 ± 1.94 µm). Most notably, these cells failed to differentiate and form myotubes. Accordingly, we designated human myoblasts cultured for <7 passages as young myoblasts YM and those cultured for >10 passages as senescent myoblasts (SM).

Our laboratory also showed that the embryonic transcription factor, NANOG, reversed the hallmarks of aging and the ability of senescent myoblasts to form myotubes^26,27^. In agreement, we transduced YM with either a lentivirus encoding for ZsGreen under the p16 promoter alone (Fig. S1a); or in combination with lentivirus encoding for NANOG under a tetracycline-regulatable promoter (Fig. S1b). Both groups of cells were induced to senesce by serial passaging and then, NANOG was expressed (Fig. S1c) by treatment with doxycycline (Dox) for 12 days (SM expressing NANOG denoted as SMN). Indeed, NANOG suppressed the promoter activity of the p16^INK4a^ (cyclin-dependent kinase inhibitor 2 A, CDKN2A, or p16), which is a well-established marker for aging (Fig. S1d, e). Furthermore, NANOG expression significantly decreased the level of SA-β-gal and glycogen accumulation, well-known markers for cellular senescence (Fig. S1f–i). These results suggest that NANOG may be used as a tool to reverse the aging hallmarks of SM and examine the accompanying changes in the signaling and/or metabolism.

**Fig. 1:**
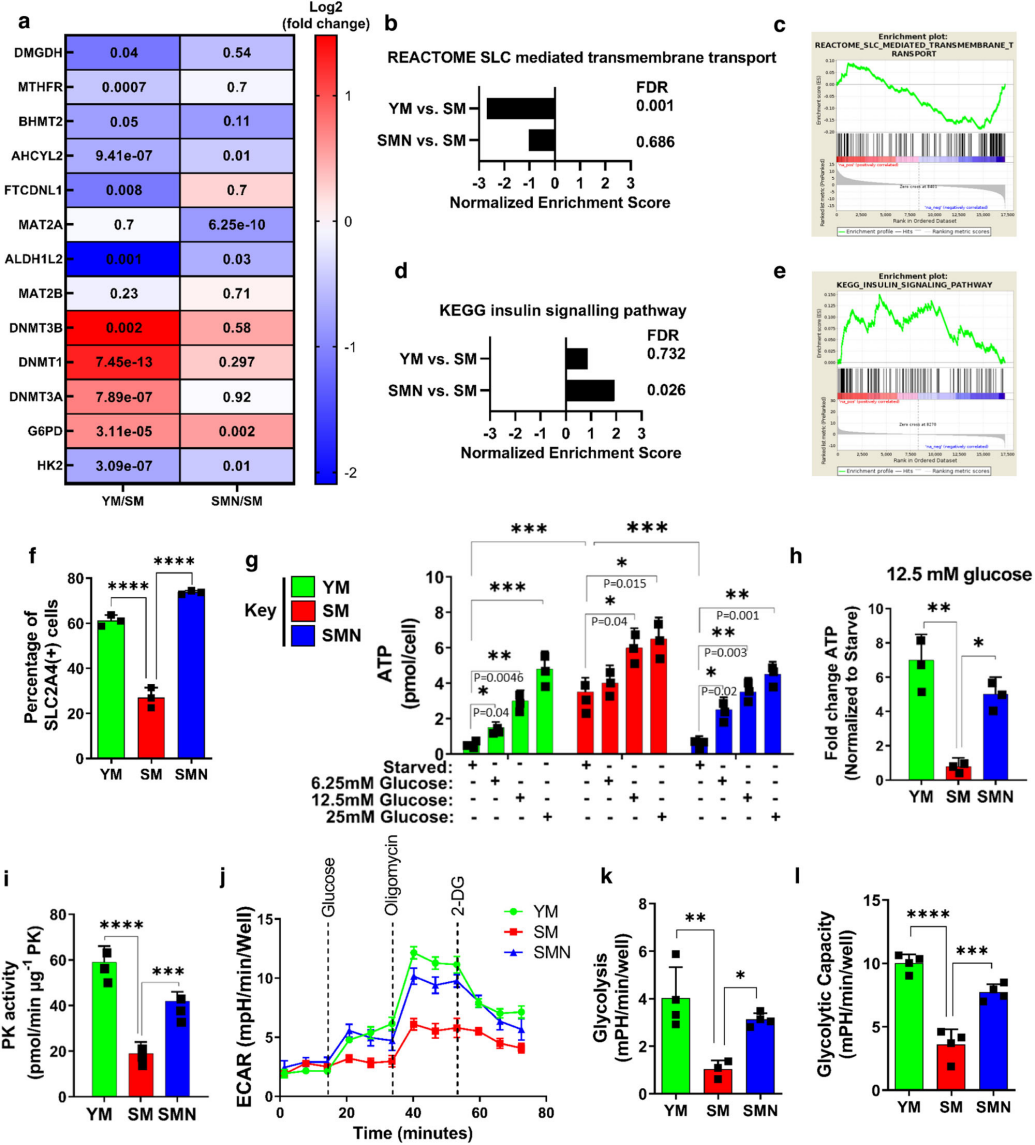
Impaired glycolytic capacity in senescent human myoblasts is restored upon NANOG expression. **a** Heat map of representative metabolic enzymes in RNA-seq with significant difference between YM and SM (YM/SM) or SMN and SM (SMN/SM). **b**–**e** GSEA analysis showing changes in SLC transporter expression and insulin signaling pathways. **f** Flow cytometry analysis of YM, SM, and SMN stained with APC-conjugated Glut4 antibody shows the percentage of SLC2A4 + cells. (****p < 0.0001) statistical significance using one-way ANOVA with Tukey’s multiple comparisons test. **g** Intracellular ATP level was measured after short-term starvation of cells for 6 h followed by treatment with the indicated glucose concentrations (6.25, 12.5, and 25 mM). (***p < 0.001) statistical significance using two-way ANOVA with Tukey’s multiple comparisons test. **h** Fold change in ATP levels upon treatment with 12.5 mM glucose as compared to no glucose control. (*p = 0.01 and **p = 0.002). **i** Pyruvate kinase act**i**vity. (***p = 0.0005 and ****p < 0.0001). **j**–**l** Measurements of extracellular acidification rate using Seahorse extracellular flux analyzer. (*p = 0.028, **p = 0.004, ***p = 0.0003, and ****p < 0.0001). For **h**–**l** plots, statistical significance using one-way ANOVA with Tukey’s multiple comparisons test. YM: green bars or green lines, SM: red bars or red lines, and SMN: blue bars or blue lines. *n* = 3 human subjects/group. Data in bar graphs are presented as mean ± SD and data in ECAR plot is presented as mean ± SEM.

RNA-sequencing showed that several genes involved in methionine metabolism and glycolysis as well as several metabolic pathways changed significantly in SM and were restored by NANOG (Fig. 1a and S2a). Gene set enrichment analysis (GSEA) revealed that solute transporters (SLC) exhibited differential expression upon senescence (SM) (Fig. 1b, c), while the insulin-signaling pathway was significantly upregulated in SMN cells (Fig. 1d, e). This result prompted us to examine the levels of SLC2A4, an insulin-dependent glucose transporter. Flow cytometry showed that as compared to the YM group, SM cells has lower levels of SLC2A4 on their surface, suggesting reduced translocation, which was restored in SMN cells (Fig. 1f and S1j). To see if there was an effect on cellular energetics, we measured intracellular ATP after short-term starvation of cells for 4 h followed by treatment with glucose at the indicated concentrations of 6.25, 12.5, and 25 mM. Surprisingly, under all treatment conditions, the maximum ATP levels were found in SM cells (Fig. 1g). However, increasing glucose concentration increased the fold change of ATP generated in YM and SMN to a much higher extent than SM (Fig. 1h). Specifically, increasing glucose from 0 to 12.5 mM increased ATP production by 10 ± 2-fold in YM and SMN but only 2.5 ± 0.5-fold in SM cells (Fig. 1h). In addition, YM and SMN cells demonstrated a dose-dependent decrease of ATP in response to 2-DG (hexokinase inhibitor, IC_50_ = 5 mM), which was not observed in the SM group (Fig. S2b). In agreement, pyruvate kinase, the enzyme that synthesizes pyruvate during glycolysis^29^, exhibited suppressed activity in SM as compared to YM and SMN cells (Fig. 1i), suggesting impaired glycolysis in SM that was restored by NANOG.

To investigate further, we employed the Seahorse glycolysis stress test to measure the extracellular acidification rate, a measure of lactate secretion. Indeed, glucose administration induced greater ECAR in YM and SMN as compared to SM cells (Fig. 1j–l). In addition, SM cells were less responsive to oligomycin (ATP synthase inhibitor, IC_50_ = 1 μM) than both YM and SMN cells. As a result, glycolysis and glycolytic capacity were significantly lower in SM cells but restored by NANOG (Fig. 1k, l).

### Rejuvenated human myoblasts repaired insulin resistance in vitro

**Fig. 2:**
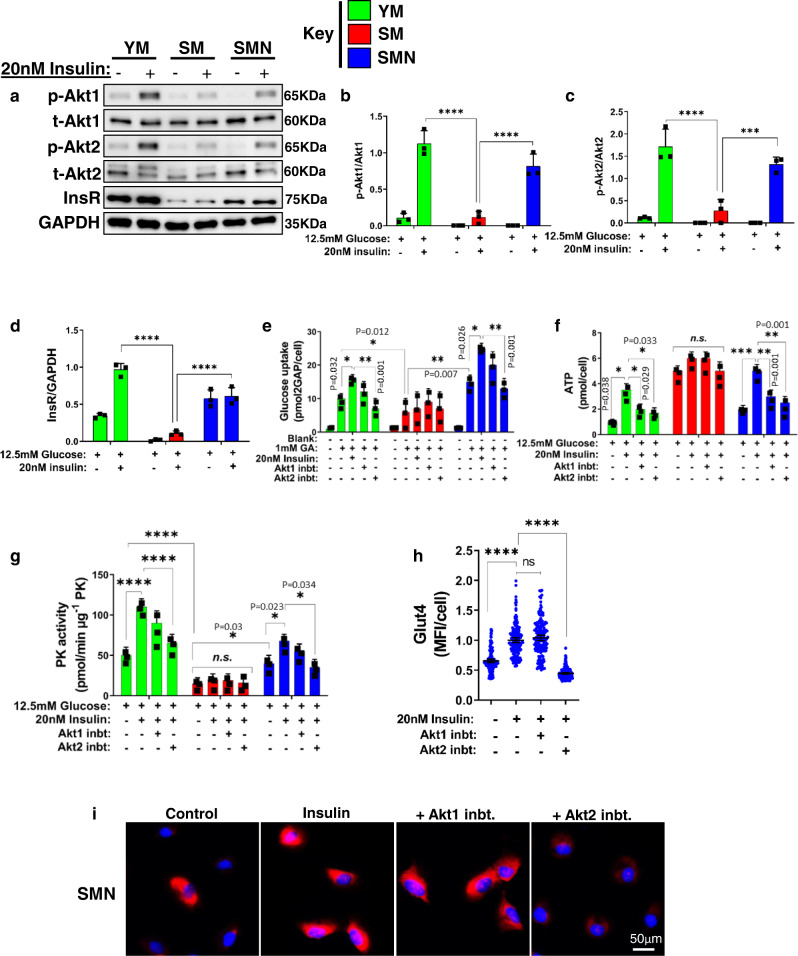
NANOG rejuvenated human myoblasts repair insulin resistance. **a** Western blots for total Akt1, Akt2, insulin receptor, and phosphorylation of Akt1 and Akt2. GAPDH served as a loading control. **b**, **c** Quantification of western blots shown as ratios of p-Akt1 to total Akt1 and p-Akt2 to total Akt2. **d** Quantification of western blots for insulin receptor normalized to GAPDH. (***p = 0.0005 and ****p < 0.0001) statistical significance using two-way ANOVA with Tukey’s multiple comparisons test. **e** Glucose uptake by myoblasts supplemented with 1 mM of glucose analog (GA), in the presence of 20 nM insulin or Akt1 or Akt2 inhibitor. **f**, **g** The levels of intracellular ATP (**f**) and pyruvate kinase activity (**g**), upon treatment with 12.5 mM glucose, 20 mM insulin, and Akt1 inhibitor or Akt2 inhibitor. For plots **e**–**g** (***p = 0.0003, ****p < 0.0001, and ns denotes *p* > 0.99) statistical significance using two-way ANOVA with Tukey’s multiple comparisons test. **h**, **i** Quantification of mean fluorescence intensity per cell (MFI/cell) (**h**) and immunostaining (**i**) of SMN for Glut4 with untreated (Control) or after treatment with insulin or insulin with Akt1 inhibitor or Akt2 inhibitor; data shown as mean ±95% CI for 150 cells per condition (scale bar = 50 µm). (****p < 0.0001 and ns denotes *p* = 0.38) statistical significance using one-way ANOVA with Tukey’s multiple comparisons test. YM: green bars or green dots, SM: red bars or red dots, SMN: blue bars or blue dots. *n* = 3 human donors/group. Data in bar graphs are presented as mean ± SD.

Since insulin-mediated activation of Akt signaling induces the translocation of SLC2A4 from cytoplasmic vesicles to the cell membrane^30^, we examined the effect of senescence on the activation of Akt and its potential role in insulin resistance. To this end, we examined the expression of total(t)-Akt1/2 and the capacity of insulin to induce phosphorylation, (p)-Akt1/2. All three groups expressed t-Akt1/2, which was phosphorylated by insulin (20 nM) in YM and SMN but not SM cells (Fig. 2a–c). At the same time, insulin receptor (InsR) levels were significantly higher in YM and SMN as compared to SM cells (Fig. 2a, d). Next, we tested whether inhibition of Akt signaling suppressed insulin-dependent glucose uptake. To this end, we supplemented the medium with 1 mM of a glucose analog (GA) that fluoresces upon phosphorylation of the achiral carbon. Glucose uptake was higher in YM and SMN cells; it further increased with insulin treatment and decreased significantly in the presence of the Akt2 selective inhibitor (CCT128930, IC_50_ = 1 μM) but not with Akt1 selective inhibitor (A-674563, IC_50_ = 1 μM) (Fig. 2e). In contrast, glucose uptake was low and was not affected by insulin treatment or Akt inhibition in SM cells, demonstrating that SM cells developed insulin resistance, which was restored by NANOG (Fig. 2e). In agreement, Akt2 inhibition decreased the stimulatory effect of insulin on ATP and PK activity in YM and SMN but not in SM cells (Fig. 2f, g). In SMN cells, insulin stimulation of SLC2A4 was averted by Akt2 inhibition but not Akt1 inhibition, suggesting that NANOG might have restored insulin sensitivity by restoring components of the Akt2 pathway (Fig. 2h, i).

### Methionine fuels ATP synthesis in human myoblasts in vitro

Since SM showed decreased glucose uptake and glycolysis, we hypothesized that senescent cells might be using a different carbon source as a fuel to meet their bioenergetic demands. To this end, we investigated amino acids and fatty acids as potential sources of excess ATP in SM cells, using β−2-chloro-alanine (GOT1/2 blocker; IC_50_ = 250 µM, an inhibitor of gluconeogenic amino acids) and etomoxir (CPT1/2 blocker; IC_50_ = 40 µM, an inhibitor of fatty acid beta-oxidation), respectively. While etomoxir had no significant effect on ATP synthesis, β−2-chloro-alanine selectively impacted ATP levels only in SM (Fig. S2c), suggesting that amino acids (AA) may be a significant energy source for senescent cells.

**Fig. 3:**
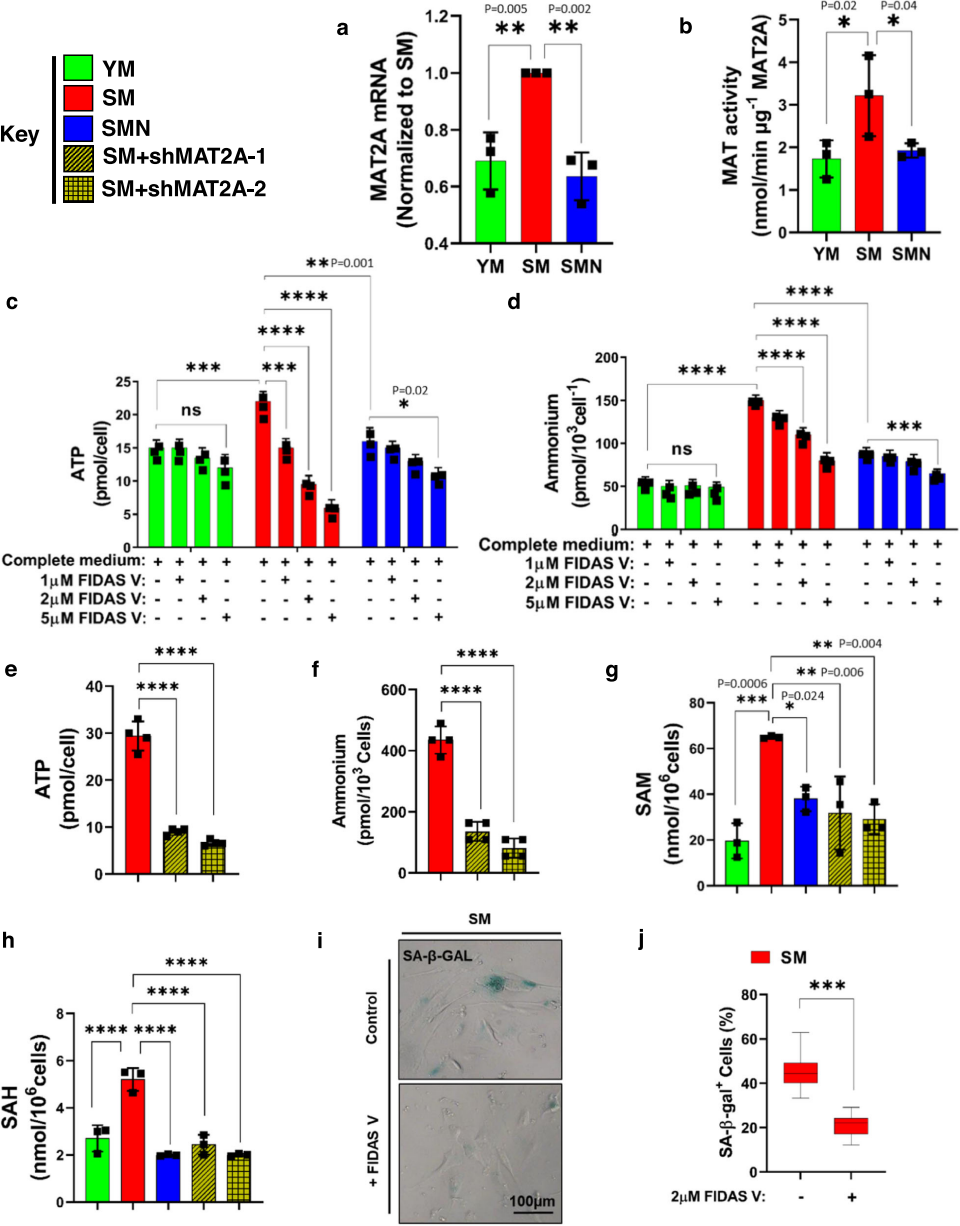
Methionine fuels ATP generation in senescent human myoblasts. **a** RT-PCR for human MAT2A expression; or **b** MAT2A activity measurements in YM, SM, and SMN cells. For **a**, **b** plots statistical significance using one-way ANOVA with Tukey’s multiple comparisons test. **c** ATP and **d** intracellular ammonium levels with the indicated concentrations of FIDAS V. **c**, **d** Statistical significance using two-way ANOVA with Tukey’s multiple comparisons test. (***p < 0.0009 and ****p < 0.0001, and ns denotes *p* > 0.98). **e** ATP and **f** intracellular ammonium levels with MAT2A knockdown using two shRNAs. **g**, **h** The level of *s*-adenosyl methionine (SAM) and *s*-adenosylhomocysteine (SAH) in human myoblast cells. For **e**–**h** plots statistical significance using one-way ANOVA with Tukey’s multiple comparisons test (****p < 0.0001). **i**, **j** Staining for SA-β-Gal in SM in the presence or absence of FIDAS V and quantification of the percentage of SA-β-Gal-positive cells (*n* = 250 cells) (scale bar = 100 μm). For the boxplots, the top and bottom lines of each box represent the 75th and 25th percentiles of the samples, respectively. The line inside each box represents the median of the samples. The upper and lower lines above and below the boxes are the whiskers. *** represents *p* < 0.001 using an unpaired *t*-test. YM: green bars, SM: red bars, SMN: blue bars, and SM + shMAT2A-1,2: yellow bars. *n* = 3 human donors/group. Data in bar graphs are presented as mean ± SD.

Furthermore, RNA-seq analysis showed increased transcripts of transporter SLC43A2 (Fig. S3a), which related to methionine uptake, suggesting that SM cells might have upregulated methionine metabolism. Indeed, both the mRNA levels and enzymatic activity of MAT2A, the rate-limiting enzyme of methionine breakdown were significantly increased in SM as compared to YM (Fig. 3a, b). Intriguingly, ectopic expression of NANOG decreased the transcription and activity of MAT2A in SMN cells, suggesting a decline in methionine catabolism (Fig. 3a, b).

To determine the role of MAT2A in SM metabolism and energy generation, we utilized FIDAS V (MAT2A selective inhibitor; IC_50_ = 1 μM) to inhibit MAT2A activity. Our results showed a dose-dependent decrease in both ATP synthesis and ammonium production upon administration of FIDAS V to SM cells only (Fig. 3c, d). While YM remained unresponsive to inhibition of MAT2A, SMN responded only to the highest concentration of FIDAS V (5μM) that decreased both ATP and ammonium by ~17 ± 2%, which was still significantly less than the 75 ± 5 % reduction measured in SM treated with the same dose.

Next, we employed two short hairpin RNAs to knock down MAT2A expression. Both shMAT2A-1 and 2 decreased MAT2A expression by 85 ± 6% and 81 ± 7%, respectively (Fig. S3b, c). Both shMAT2A-1 and -2 suppressed ATP, and ammonium production significantly **(**Fig. 3e, f), in agreement with chemical inhibition. Notably, MAT2A knockdown decreased *S*-adenosyl methionine (SAM) and *S*-adenosylhomocysteine (SAH) production (Fig. 3g, h), as well as SA-β-gal expression (Fig. 3i, j) and glycogen accumulation (Fig. S3d) in SM cells.

### Inhibiting MAT2A restores glycolysis in progeric mouse muscle cells

The Lamin A Knocked In (LAKI) mouse model of progeria carries a mutation in the lamin A gene (*lmna*^G609G/G609G^) resulting in accumulation of the truncated form of lamin A (progerin), which causes premature aging, as evidenced by an increased number of senescent cells, progressive skeletal muscle atrophy, weight loss, abnormal Posture, marked curvature of the spine and significantly shorter life span (103 days as compared to more than 2 years for wild-type mice)^31^. To examine the effects of NANOG in progeric skeletal muscle, we crossed LAKI mice (L) and Tet-On-NANOG mice to give rise to progeny with a fast-aging progeria phenotype capable of NANOG expression upon doxycycline (Dox) administration (LN). The animals used in this study expressed NANOG under the tetracycline-inducible promoter (TetO) from the Col1 locus and the reverse tetracycline transactivator (rtTA) from the ROSA26 locus.

**Fig. 4:**
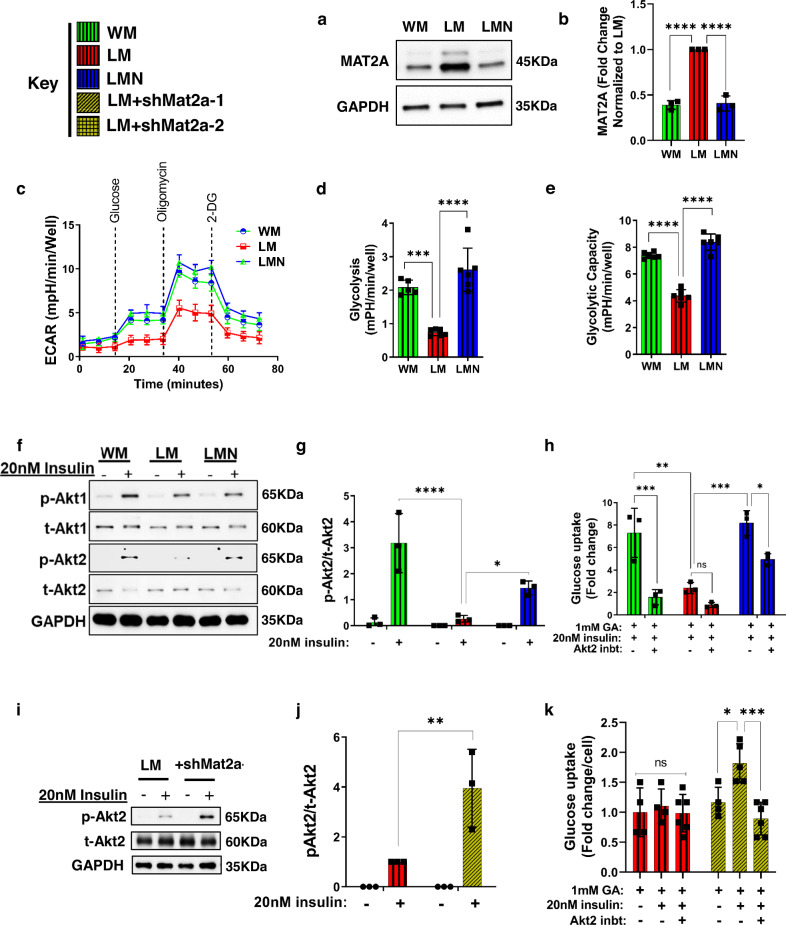
Rejuvenated progeric mouse myoblasts regained glycolytic capacity and insulin sensitivity. **a**, **b** Western blots and quantification for MAT2A in mouse myoblasts (Wild-type myoblast: WM, LAKI myoblast: LM, LAKI-NANOG myoblast: LMN). GAPDH served as a loading control. **c**–**e** Measurements of extracellular acidification rate (ECAR) and calculation of glycolysis and glycolytic capacity from ECAR. Data in the ECAR plot is presented as mean ± SEM. For **b**, **d**, and **e** plots statistical significance using one-way ANOVA with Tukey’s multiple comparisons test (***p = 0.0002 and ****p < 0.0001). **f** Western blots of total (t) and phosphorylated (p) Akt1 and Akt2; GAPDH served as a loading control. **g** Densitometric quantification of western blot showing the ratio of p-Akt2 to t-Akt2 in mouse myoblasts. (*p = 0.032 and ****p < 0.0001) statistical significance using two-way ANOVA with Sidak’s multiple comparisons test. **h** Glucose uptake in response to insulin and Akt2 inhibition. (*p = 0.04, **p = 0.002, ***p = 0.0004, and ns re*p*resents *p* = 0.83) statistical significance using two-way ANOVA with Sidak’s multiple comparisons test. **i**, **j** Western blot and densitometric analysis of p-Akt2 to t-Akt2 in LM in response to insulin and Mat2a-1 knockdown. (**p = 0.007) statistical significance using two-way ANOVA with Tukey’s multiple comparisons test. **k** Glucose uptake in response to insulin and Akt2 inhibition in control and shMat2a-1 LM cells. (*p = 0.04, ***p = 0.0007, and ns re*p*resents *p* = 0.99) statistical significance using two-way ANOVA with Tukey’s multiple comparisons test. WM: green bars or green lines, LM: red bars or red lines, LMN: blue bars or blue lines, and LM + shMat2a-1: yellow bars. *n* = 5 animals per cohort. Data in bar graphs are presented as mean ± SD and data in ECAR plot is presented as mean ± SEM.

We hypothesized that LAKI myoblasts (LM) rewired their metabolism to use methionine to compensate for impaired glycolysis and that NANOG corrected this aberration. To test this hypothesis, we isolated myoblasts from LN skeletal muscle that could express NANOG upon treatment with Dox (LMN) (Fig. S3e) and wild-type mouse myoblasts WM from age-matched 2–3-month-old mice. The level of MAT2A was greater in LM cells as compared to WM and LMN (Fig. 4a, b). Furthermore, LM cells exhibited marked depletion of *slc2a4* transcripts that were partially restored in LMN cells (Fig. S4a), prompting us to examine glycolysis by measuring ECAR. Indeed, LM cells showed decreased glycolysis and glycolytic capacity that was restored in LMN (Fig. 4c–e). Furthermore, stimulation with insulin led to the phosphorylation of Akt1 to a similar extent in all three groups (Fig. 4f and S4b). On the other hand, insulin phosphorylated Akt2 in WM and LMN, but failed to do so in LM cells, implicating Akt2 signaling in the insulin resistance of progeria myoblasts (Fig. 4f, g). Indeed, upon insulin stimulation glucose uptake increased in WM and LMN, but not in LM cells; while the Akt2 inhibitor suppressed insulin-induced glucose uptake in WM and LMN cells, LM cells remained unresponsive (Fig. 4h).

Next, we designed and confirmed that shMat2a-1 and shMat2a-2 decreased MAT2A expression by 79 ± 9% and 90 ± 5%, respectively (Fig. S4c, d). Similar to SM, the PK activity of LM cells was significantly suppressed (Fig. S4e) but increased by 2.1- and 3.75-fold by shMat2a-1 and shMat2a-2, respectively (Fig. S4f). Furthermore, knocking down MAT2A restored the capacity of insulin to phosphorylate Akt2 in LM cells (Fig. 4i, j). Indeed, insulin stimulation increased glucose uptake in LM cells expressing shMat2a-1, but not in LM control; while the Akt2 inhibitor, CCT128930 suppressed insulin-induced glucose uptake only in shMat2a cells (Fig. 4k).

### MAT2A inhibition restores the differentiation capacity of mouse myoblasts in vitro

**Fig. 5:**
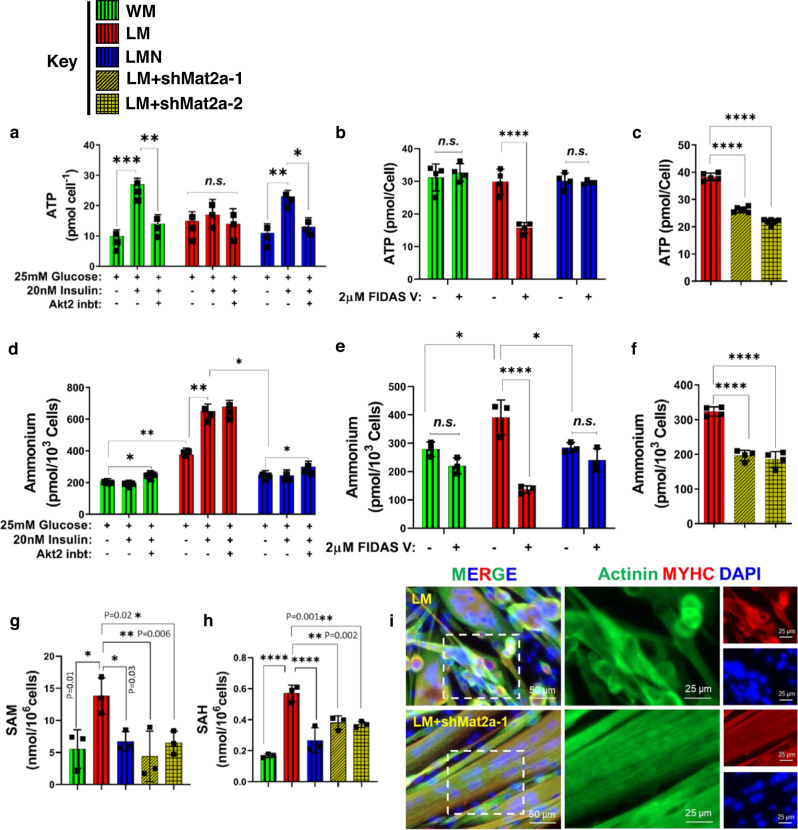
Inhibiting MAT2A decreases ammonium levels and restores differentiation capacity in progeric mouse myoblasts. **a** ATP levels in response to insulin and Akt2 inhibition. (*p = 0.02, **p = 0.00076, ***p = 0.0002, and ns represents *p* > 0.37) statistical significance using two-way ANOVA with Tukey’s multiple comparisons test. **b** ATP levels in response to MAT2A inhibitor FIDAS V. **c** ATP levels in LM cells with Mat2a knockdown. **d** Ammonium levels in response to insulin and Akt2 inhibition. (*p < 0.04 and **p < 0.001) statistical significance using two-way ANOVA with Tukey’s multiple comparisons test. **e** Ammonium levels in response to MAT2A inhibitor FIDAS V. For plots **b** and **e** statistical significance using two-way ANOVA with Sidak’s multiple comparisons test. (*p < 0.04, ****p < 0.0001, and ns represents *p* = 0.99). **f** Ammonium levels in LM cells with Mat2a knockdown. For **c** and **f** plots statistical significance using one-way ANOVA with Tukey’s multiple comparisons test (****p < 0.0001). **g**, **h** Level of *s*-adenosylmethionine (SAM) and *s*-adenosylhomocysteine (SAH) at the indicated conditions. Statistical significance using one-way ANOVA with Dunnett’s multiple comparisons test (****p < 0.0001). **i** Immunostaining for myosin heavy chain (MyHC) (red) and actinin (green) show**i**ng skeletal muscle differentiation (scale bar = 50 µm). WM: green bars, LM: red bars, LMN: blue bars, and LM + shMat2a-1,2: yellow bars. *n* = 5 animals per cohort. Data in bar graphs are presented as mean ± SD.

In WM and LMN, insulin (20 nM) stimulation increased ATP production, which decreased upon Akt2 inhibition (Fig. 5a). However, LM resisted insulin stimulation and subsequent Akt2 inhibition failed to suppress ATP production (Fig. 5a). On the other hand, both 2 μM FIDAS V or MAT2A knockdown decreased ATP synthesis in the LM group significantly (Fig. 5b, c). As compared to WM and LMN, ammonium production was higher in LM and further upregulated by 20 nM insulin (Fig. 5d). Intriguingly, FIDAS V (Fig. 5e) or shMat2a-1 and -2 (Fig. 5f) suppressed ammonium production only in LM cells. Additionally, in comparison to WM, the mRNA levels for ammonium transporter, *slc42a2* was also higher in LM cells and decreased by NANOG (Fig. S4g). These results suggested that methionine catabolism might be higher in LM, perhaps as a result of loss of Akt2 activity. Indeed, the level of SAM and SAH, a major metabolite in the methionine pathway that is known to impair skeletal muscle integrity^32,33^, was significantly increased in LM and decreased by NANOG or MAT2A knockdown (Fig. 5g, h).

Further, LM cells failed to differentiate into myotubes in vitro; instead, they fused into disorganized globular structures incapable of synchronized contraction. Upon expression of NANOG or MAT2A knockdown, progeric mouse myoblasts fused into aligned striated myotubes that exhibited Z-bands and regained contraction ability (Fig. 5i, S5a, and Supplementary Videos 1–4).

### NANOG restores markers of methionine catabolism and glycolysis in the TA muscle of LAKI mice

Since NANOG reversed many of the metabolic indicators of progeria cells we tested whether NANOG could have similar effects in vivo. To avoid potential systemic effects^34,35^, we induced NANOG expression in the skeletal muscle of progeria mice using a polymeric delivery vehicle (Elvax), which was wrapped around the TA and EDL muscles and enabled sustained release of Dox over a period of two weeks^36^. In the right TA, Elvax (100 μl) was loaded with Dox (25 mg) to induce NANOG expression, while the left limb served as an internal control (Elvax, no Dox). Two weeks later, the Elvax was removed surgically from both legs and muscle function was evaluated 3 weeks later.

**Fig. 6:**
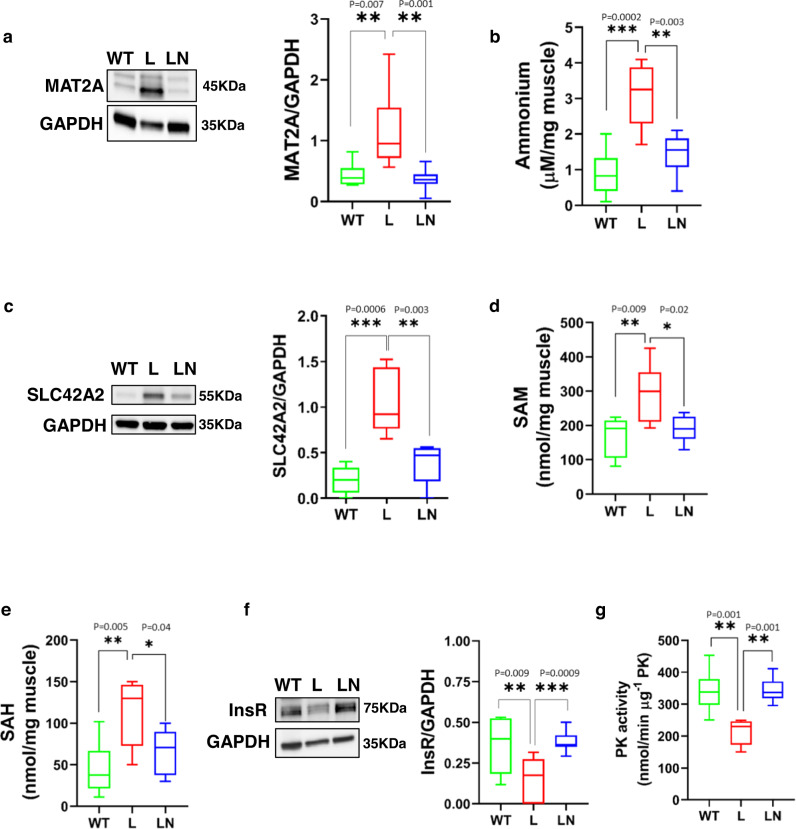
NANOG restores markers of methionine catabolism and glycolysis in the TA muscle of LAKI mice. Elvax (100 μl) loaded with Dox (25 mg) was implanted around the right TA muscle of LAKI mice to induce NANOG expression, while the left limb served as an internal control (Elvax, no Dox). Two weeks later, the Elvax was removed and the TA muscle was evaluated 3 weeks later. **a** Western blot and band quantification for MAT2A; GAPDH served as loading control **b** Ammonium levels. **c** Western blot and band quantification for SLC42A2; GAPDH served as loading control. **d**, **e** Effect of NANOG on *s*-adenosyl methionine (SAM) and *s*-adenosylhomocysteine (SAH) levels. **f** Western blot and band quantification for InsR; GAPDH served as loading control. **g** PK activity levels. WT: green boxes, L: red boxes, and LN: blue boxes. *n* = 5 animals per cohort. For the boxplots, the top and bottom lines of each box represent the 75th and 25th percentiles of the samples, respectively. The line inside each box represents the median of the samples. The upper and lower lines above and below the boxes are the whiskers. Statistical significance using one-way ANOVA with Tukey’s multiple comparisons test.

Similar to cultured myoblasts, the TA muscle of L mice showed significant upregulation of MAT2A expression (Fig. 6a), elevated ammonium concentration (Fig. 6b), greater levels of the ammonium transporter, SLC42A2 (Fig. 6c), increased SAM and SAH production (Fig. 6d, e), suppressed insulin receptor expression (Fig. 6f), and impaired PK activity (Fig. 6g); all of which were reversed by Dox administration (LN).

### NANOG restores skeletal muscle regeneration after cardiotoxin injury to TA of LAKI mice

**Fig. 7:**
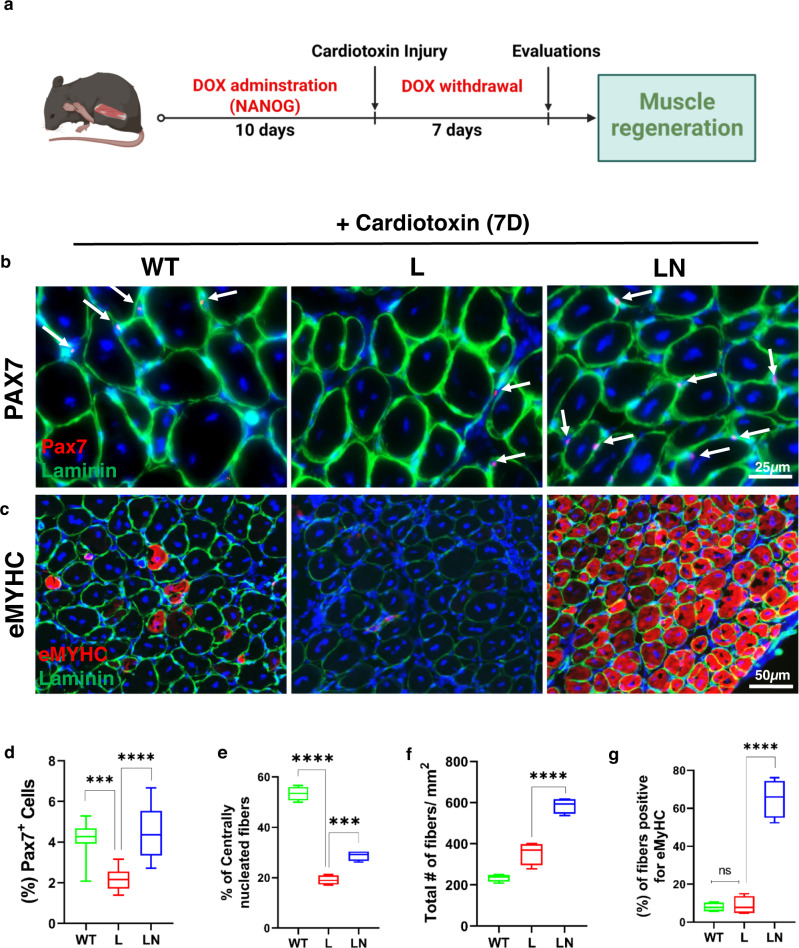
NANOG restores skeletal muscle regeneration of LAKI mouse muscle in vivo. **a** Graphic representation of the schedule for CTX injection and the experimental procedure. **b**, **c** Immunostaining for **b** Pax7 (red) and laminin (green) (Scale bar = 25 μm); **c** eMyHC (red) and laminin (green) (Scale bar = 50 μm) after 7 days of cardiotoxin injury. **d** Quantification of % Pax7+ cells. **e** Quantification of the number of centrally nucleated myofibers with more than two nuclei. **f**, **g** Quantification of the total number of fibers **f** and number of fibers positive for eMyHC **g**. WT: green boxes, L: red boxes, and LN: blue boxes. *n* = 4 animals per cohort. For the boxplots, the top and bottom lines of each box represent the 75th and 25th percentiles of the samples, respectively. The line inside each box represents the median of the samples. The upper and lower lines above and below the boxes are the whiskers. Statistical significance using one-way ANOVA with Tukey’s multiple comparisons test (***p < 0.001, ****p < 0.0001, and ns represent *p* = 0.98).

To assess the impact of NANOG on skeletal muscle regeneration, we utilized cardiotoxin (CTX) to induce injury. After 10 days of NANOG expression, the TA muscle was injured by intramuscular injection of CTX and 7 days later harvested for evaluation of tissue regeneration (Fig. 7a). Immunostaining for myogenic transcription factor (Pax7), showed diminished numbers of Pax7+ cells (Fig. 7b, d) and significant attrition in multinucleated fibers (Fig. 7c, e) of the TA of L as compared to WT mice. Notably, Dox administration increased the number of both Pax7+ and multinucleated fibers in LN mice (Fig. 7b, d, e). We also quantified the number of myofibers that were positive for embryonic myosin heavy chain positive (eMyHC+), an indicator of newly formed myotubes. Dox treatment induced a significant increase in total numbers of myofibers (Fig. 7c, f) and eMyHC+ fibers in LN mice (Fig. 7c, g), indicating significantly enhanced muscle regeneration with NANOG.

### NANOG restores strength to the muscle of LAKI mice

**Fig. 8:**
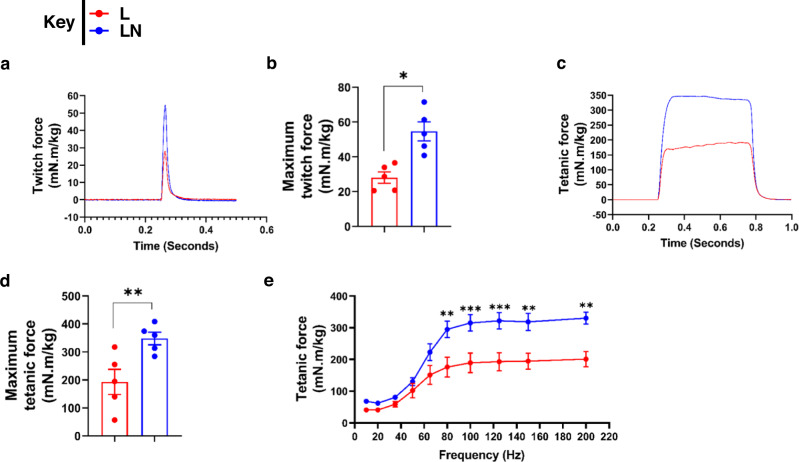
NANOG restores contraction force generation by LAKI mouse muscle in vivo. **a**, **b** Effect of NANOG on twitch force contraction. **c**, **e** Effect of NANOG on tetanic force contraction profile **c**, maximum tetanic force **d**, and tetanic force as a function of stimulation frequency **e** by LAKI dorsiflexor muscles in vivo. L: red bars or red lines, and LN: blue bars or blue lines. *n* = 5 animals per cohort. Data in bar graphs are presented as mean ± SD. Plot **b**, **d** *p = 0.02 and **p = 0.007 using unpaired *t*-test. Plot **e**, statistical significance using two-way ANOVA with Sidak’s multiple comparisons test (***p < 0.001 and ****p < 0.0001).

Immunostaining for laminin showed a higher percentage of myofibers with an area >1000 µm^2^ in the TA muscle of the LN group (Fig. S6a, b). Using a force transducer, we measured the aggregate torque produced by the dorsiflexor muscles of live mice. Specifically, we measured the twitch force, maximum isometric tetanic force, and force–frequency relationship generated by the right limb that received Dox as compared to the left control limb. Notably, NANOG significantly increased the twitch force by 94% (*n* = 5, *p* < 0.05) (Fig. 8a, b) and tetanic force by 80% (*n* = 5, *p* < 0.01) (Fig. 8c–e).

Following the in vivo measurements, fast-twitch EDL muscle was harvested from each mouse and isometric tension was measured in an isolated tissue bath in response to electrical stimulation. EDL contraction force in LAKI mice decreased threefold compared to wild-type control mice. Similar to in vivo measurements, ex vivo force measurements showed that NANOG increased the isometric contraction of progeric EDL muscle by 54% (*n* = 7, *p* < 0.05) (Fig. S6c).

### Inhibition of MAT2A restores the function of LAKI skeletal muscle in vivo

Next, we examined whether the effects of NANOG on skeletal muscle function in vivo were, at least in part, due to the inhibition of MAT2A. To this end, we employed Elvax (100 μl) loaded with FIDAS V (25 mg) to block MAT2A in vivo.

**Fig. 9:**
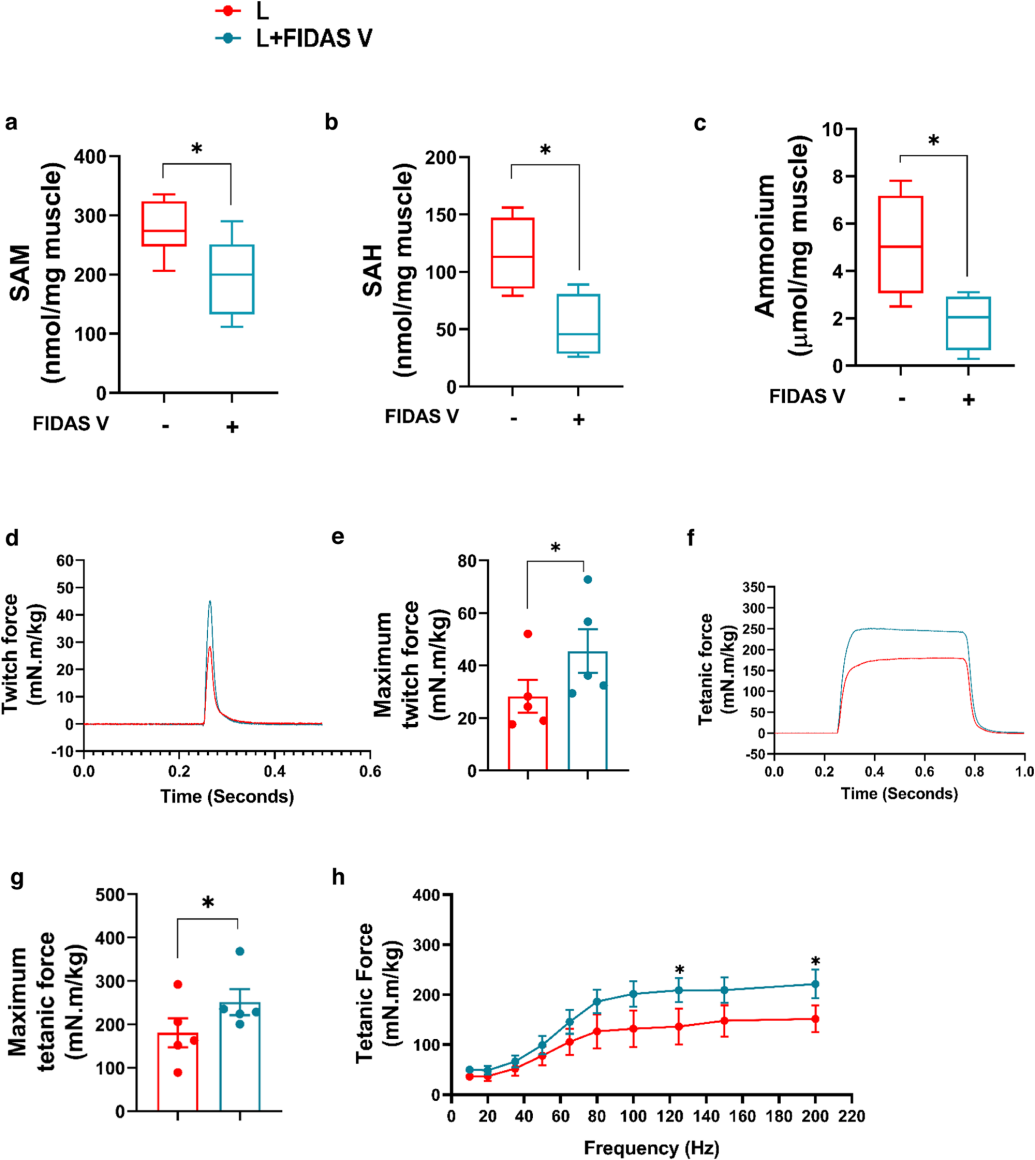
MAT2A inhibition increases contraction force generation by LAKI mouse muscle in vivo. **a**–**c** Effect of FIDAS V on *s*-adenosyl methionine (SAM), *s*-adenosylhomocysteine (SAH), and ammonium production in TA muscle. Statistical significance using unpaired *t*-test (Plot a; *p = 0.03, b: *p = 0.02, c: *p = 0.04). **d**, **e** Effect of FIDAS V on twitch force *p = 0.046 using paired *t*-test. **f**–**h** Effect of FIDAS V on tetanic force contraction profile (**f**), maximum tetanic force (*p = 0.019 using paired *t*-test) (**g**), and tetanic force as a function of stimulation frequency (*p = 0.04 using two-way ANOVA with Tukey’s multiple comparisons test) (**h**) by LAKI dorsiflexor muscles in vivo. L are red bars or red lines, and L + FIDAS V are light blue bars or light blue lines. *n* = 5 animals per cohort. Data in bar graphs are presented as mean ± SD and for the boxplots, the top and bottom lines of each box represent the 75th and 25th percentiles of the samples, respectively. The line inside each box represents the median of the samples. The upper and lower lines above and below the boxes are the whiskers.

Treatment with FIDAS V demonstrated comparable trends as NANOG. FIDAS V decreased both SAM and SAH (Fig. 9a, b) and also ammonium production (Fig. 9c), and increased both twitch force by 60% (*n* = 5, *p* = 0.053) (Fig. 9d, e) and tetanic force by 40% (*n* = 5, *p* < 0.05) (Fig. 7f–h). Interestingly, administration of FIDAS V increased the isometric tension of LAKI EDL by ~2-fold compared to LAKI control muscle (*n* = 4, *p* < 0.05) (Fig. S6d). Collectively, these results suggest that inhibiting the MAT2A, even for 2 weeks had significant effects on muscle metabolism and physiology reversing the age-related loss of function.

## Discussion

In this study, we provide evidence that both senescent human myoblasts and progeric mouse myoblasts experience loss of Akt2 signaling and glycolysis and employ methionine for energy production. Human senescent and mouse progeria myoblasts showed diminished levels of the insulin receptor, and significantly decreased glucose uptake, and insulin resistance, yet SM cells produced higher levels of energy. While glucose treatment increases ATP in all cells including SM, the fold change was much lower in SM as compared to YM or SMN cells (Fig. 3). Although the level of ATP was not highest in LM, the contribution of MAT2A to ATP levels was greater, as blocking MAT2A decreased ATP in LM but not in WM or LMN (Fig. 5). Therefore, in both human and mouse cells, decreased glycolysis was accompanied by activation of methionine catabolism, possibly as a way to restore bioenergetics. Indeed, inhibition of glycolysis, fatty acid oxidation or amino acid catabolism pathways showed that while young cells produced less ATP upon inhibition of glycolysis, senescent cells responded only to the amino acid inhibitor, β-2-chlorolanine (Fig. S2c).

**Fig. 10:**
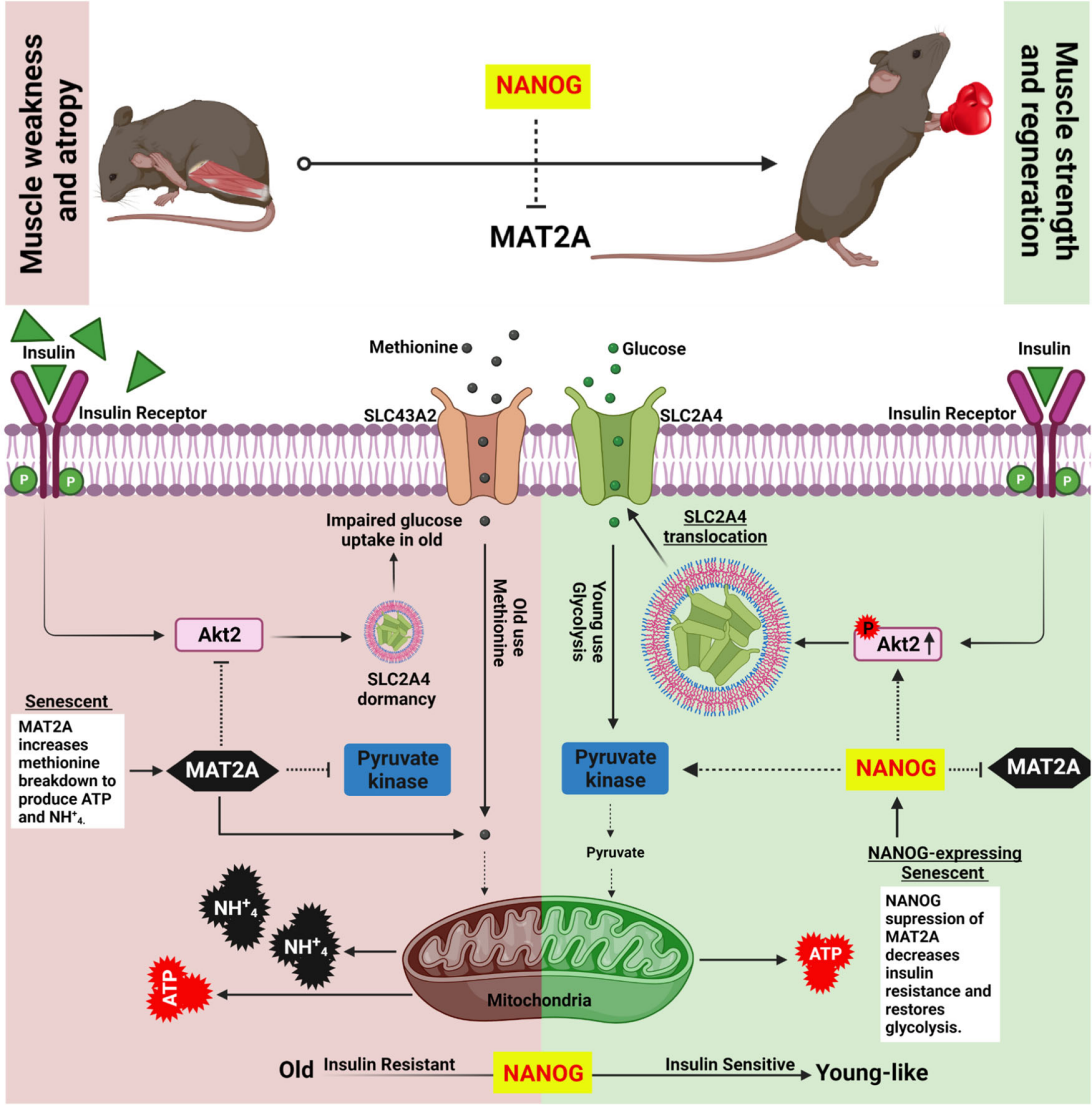
Schematic of the metabolic and signaling pathways affected in senescent or progeria myoblasts and reversed by NANOG or MAT2A inhibition. Both insulin resistance and Akt2 inactivation prevent GLUT4 translocation and inhibit glucose usage for energy production. In response, bioenergetics is maintained and augmented by MAT2A catalyzing the breakdown of methionine for ATP. Ammonium generation is a side effect of methionine catabolism that may contribute to weakness and atrophy of aged muscle. NANOG expression restores insulin sensitivity, muscle regeneration, and strength via suppression of MAT2A.

In agreement, human senescent and mouse progeric myoblasts and LAKI mouse skeletal muscle showed significant upregulation of MAT2A, the enzyme that catalyzes the first reaction in methionine catabolism. Conversely, blocking MAT2A either chemically or via shRNA decreased ATP production, while blocking glycolysis with 2-DG had no effect, suggesting that senescent cells use methionine to produce ATP. Interestingly, the transcription factor NANOG has been shown to reverse the hallmarks of aging in senescent myoblasts^26^, reinstated glycolysis, and decreased the levels of MAT2A similar to young myoblasts, indicating that such metabolic rewiring to a pre-senescent state may be feasible (Fig. 10).

Typically, skeletal muscle cells possess an evolutionary ability to utilize MAT2A for methionine breakdown into various catabolites including glutathione, MNA, *s*-adenosylhomocysteine, and taurine^20,37,38^. However, the use of MAT2A for ATP production has not been reported, especially in the context of aging, where cells seem to be rewiring their metabolic networks to enhance survival. Seemingly, MAT2A was upregulated by exhaustive exercise^39^, which perhaps mimics starvation. Although previous studies showed that MAT2A alters lipid metabolism^17^, we found that etomoxir, a CPT inhibitor, and 2-DG, a glycolysis inhibitor failed to impact ATP levels in senescent myoblasts (Fig. S2c).

The insulin receptor (IR) is a hetero-tetrameric complex, consisting of two extracellular α/β subunits^40^. Insulin binding to IR then induces the β subunits to undergo autophosphorylation at adjoining tyrosine residues^41^. The activated IR then phosphorylates intracellular substrates that include the insulin receptor substrate family (IRS1-4), Gab-1, Cbl, and Shc isoforms^41^. IR regulates glucose metabolism through Akt, mTOR, and AMP kinase^40^. We discovered low levels of IR in LAKI TA muscle, suggesting that insulin signaling might be impaired. Insulin signaling regulates muscle regeneration^42^ and glucose^43^ metabolism via an Akt-dependent mechanism. Akt1 and Akt2 isoforms, which are highly expressed in skeletal muscle^42^, have distinct but overlapping functions^42,43^. Akt2 controls insulin-stimulated glucose metabolism and in skeletal muscle, it exerts greater control over insulin-mediated redistribution of GLUT4 from cytosolic to membrane localization^42^. Further, insulin activates Akt2 to induce subcellular redistribution of AS160 substrate, which assembles and translocates GLUT4-loaded vesicles^43^. On the other hand, Akt1 was reported to amplify insulin signaling rather than control the insulin response^44,45^. Notably, the higher levels of ATP, impaired Akt2 signaling, and low pyruvate kinase activity in human senescent and mouse progeria myoblasts were reversed by inhibition of MAT2A, suggesting that increased methionine catabolism might be affecting glycolysis.

Interestingly, the level of ammonium, a byproduct of amino acid deamination, was significantly higher in LM and muscle tissue of progeric mice and decreased by NANOG expression. Ammonium buildup has been associated with loss of cellular function and skeletal muscle atrophy^19,46,47^, which has also been linked to the development of insulin resistance and diabetes^48–52^. Indeed, our results indicate that senescent myoblasts showed signs of insulin resistance as they lost the ability to phosphorylate Akt2 in response to insulin. Notably, ammonium levels, insulin sensitivity, and glucose uptake were restored by knocking down or inhibiting MAT2A, suggesting that increased use of methionine might be contributing to insulin resistance. Since NANOG suppressed MAT2A levels, these results suggest that the rejuvenating effects of NANOG might be mediated, at least in part, via the downregulation of MAT2A. These findings may explain the results of recent studies implicating methionine to inflammation, decreased lifespan, and others that touted the benefits of MR^17,22,53–55^ in improving insulin sensitivity^19,21^ and restoring glycemic control^56,57^. Indeed, MR resulted in decreased inflammation^17^, increased expression of DNA-damage response genes^17^, and significant improvement in the lipid profile^17^ of progeric mice.

As expected, progeric mice exhibited a significant impairment in muscle regeneration following CTX injury. Specifically, LAKI muscle contained a reduced number of Pax7+ cells and centrally nucleated fibers with diminished eMyHC expression at 7 days post-injury as compared to WT animals. Notably, NANOG restored the percentage of Pax7+ cells, and increased the total number of myofibers as well as the percentage of eMyHC+ myofibers significantly, suggesting that NANOG enhanced the regenerative capacity of progeric muscle. In agreement with diminished regeneration, the ability of the fast-twitch EDL muscle of LAKI mice to generate force decreased threefold as compared to control subjects. Most notably, NANOG expression for two weeks completely restored the force generated by LAKI, which correlated with a higher fraction of larger muscle fibers in control animals (Fig. S6). Treatment with FIDAS V also increased the force significantly by ~40%, albeit to a lesser extent than NANOG (~2-fold). Such a remarkable increase in force generation induced by either NANOG or FIDAS V may reflect the combination of enhanced glucose uptake/glycolysis and decreased ammonium toxicity due to the restriction of methionine usage by muscle cells.

Senescent human myoblasts and progeria myoblasts, rewire their metabolism from glycolysis to amino acid and specifically, methionine catabolism, as a way to maintain or even increase ATP levels. This comes at the expense of the accumulation of toxic byproducts including ammonium that can damage DNA and impair cellular function. In addition, upregulation of MAT2A induced insulin resistance, possibly implicating methionine usage to the development of type II diabetes with aging. Notably, NANOG decreased MAT2A expression and ammonium levels, restored glycolysis, the regeneration capacity after CTX injury, and the force-generating ability of the aged muscle. Similarly, MAT2A inhibition restored insulin sensitivity, glycolysis, and ammonium levels and enhanced the force-generating ability of aged mice significantly, suggesting a possible link between methionine metabolism and the regenerative capacity of skeletal muscle.

## Methods

### Generation of LAKI-NANOG transgenic mice

The Nanog transgenic mice, carrying the M2-rtTA gene inserted within the Rosa26 allele and a cassette containing the Nanog cDNA under the control of the Dox-responsive promoter (tetO) inserted downstream of the Col1a1 locus (C57BL/6; Col1a1 tetO-Nanog/+; ROSA26rtTA/rtTA), were provided by the laboratory of Dr. Manuel Serrano at The Barcelona Institute of Science and Technology, Spain^34^. The mouse model carrying LMNA mutation G609G (LAKI, C57BL/6, LmnaG609G/G609G) were generated by Carlos López-Otín at the University of Oviedo, Spain^31^ and donated by Dr. Dudley Lamming at the University of Wisconsin. To generate a fast-aging progeria mouse model which is capable of expressing NANOG upon Dox administration, LAKI and NANOG transgenic mice were crossed by in vitro fertilization at the Roswell Park Gene Targeting and Transgenic Shared Resource, Buffalo, NY to obtain the LAKIN genotype (Col1a1 tetO-Nanog/+; ROSA26rtTA/rtTA, LmnaG609G/+ or LmnaG609G/G) and their control littermates (Col1a1+/+; ROSA26rtTA/rtTA, LmnaG609G/+, or LmnaG609G/G). In vitro fertilization using the method from the Center for Animal Resources and Development (CARD) was done as described previously^58^. Mice were genotyped by polymerase chain reaction (PCR) of tail DNA using the primers located in Supplementary Table 1 for DNA amplification.

The experiments were performed with both male and female mice: For the cardiotoxin injury model, WT: two males and two females, L: three males and one female, LN: two males and two females. For in vitro force measurements, WT: four males and two females, L: three males and three females, LN: three males and three females, L + FIDAS V: four male mice. For in vivo force measurements: L: one male and four females, LN: one male and four females, L + FIDAS V: five male mice. Experiments commenced the homozygous progeria mice (LmnaG609G/G) were 3 months old. Aging was confirmed by the appearance of signs of premature aging, such as kyphosis, muscle loss, weight loss, and gray hair. The mice were housed with a 12 h light/dark cycle between 6:00 and 18:00 in a temperature-controlled room (22 °C) with free access to food and water. Humidity is to be between 30–70%. All animal research protocols were approved by the Institutional Animal Care and Use Committee (IACUC) of the University at Buffalo in compliance with the Animal Welfare Act, Public Health Service Policy on humane care and use of laboratory animals, and other federal statutes and regulations relating to animals and experiments involving animals.

### Polymeric drug delivery in mouse dorsiflexor muscles

Elvax 40 W beads were prepared as described previously^59^. Briefly, Elvax beads were washed three to four times in 95% ethanol for one week with continuous stirring and then dried on filter paper. Next, 100 mg of beads were dissolved in 900 μl of methylene chloride in a glass culture tube. Doxycycline (25 mg/100 µl), FIDAS V (25 mg/100 µl), or saline was dissolved in 1% fast green in DMSO and the elvax mixture was vortexed at medium speed for 3 min and poured onto a glass slide with a piece of parafilm cut to form a spacer. The slide was placed on powdered dry ice and a second slide was clamped on top of the first slide. The elvax was transferred to −70 °C for 2–5 days and then moved to −20°. To implant the elvax, mice were anesthetized on ice until a surgical plane of anesthesia occurred when body temperature reached 12–16 °C. A small incision was made in the lateral skin of the distal hindlimb and the fascial plane between the anterior and posterior compartments was cut. A 3 × 4 mm rectangle of Elvax was placed bilaterally between the two compartments and on top of tibialis (TA) muscle. The incision was sutured and mice were returned. Drug-impregnated elvax was placed in one leg and control Elvax (no drug) was placed in the opposite leg. The elvax was left in place for 2 weeks. FIDAS V was reconstituted per the manufacturer’s recommendation in DMSO using a solubility factor of 100 mg/mL to make a stock solution.

### Mouse muscle regeneration model and cardiotoxin injection

Muscle injury in WT, L, or LN mice was induced by injecting cardiotoxin (CTX). Cardiotoxin (CTX, from Naja mossambica mossambica; Sigma) that was dissolved in sterile saline at a concentration of 20 μM. Tibialis anterior (TA) muscles of 2.5-month-old mice were injected with 50 μl CTX and harvested at 7 days post-injury.

### Ex vivo contractile measurements

After 3 weeks of Elvax withdrawal, mice were individually euthanized by intraperitoneal injection of ketamine/xylazine (200 mg/kg and 20 mg/kg, respectively). The extensor digitorum longus (EDL) were dissected and their in vitro contractile properties were measured using a previously described protocol^60^. In brief, the dissected muscle bundles were mounted between large platinum-stimulating electrodes and placed within the external chamber of a jacketed bath containing Krebs solution (6.5 mM d-Glucose anhydrous, 2 mM CaCl2, 12 mM NaHCO3, 1 mM KH2PO4.H2O, 1 mM MgCl2, 4 mM KCl, and 137 mM NaCl) maintained at 27 °C and constantly perfused by a 95% O_2_–5% CO_2_ mixture. Isometric force measurements were obtained with a force transducer mounted on a vertically movable stand. The muscle bundle was then sequentially stimulated at frequencies of 10, 20, 35, 50, 65, 80, 100, 125, 150, and 200 Hz to obtain a force–frequency curve. Data were acquired and analyzed using Spike 2 software (Windows, version 6, CED Products). On completion of the measurements, the muscle bundle was removed from the apparatus, it was blotted dry, and its weight was measured on an analytic balance.

### In vivo contractile measurements

Mice are anesthetized using isoflurane (2% isoflurane, 0.6% oxygen). Subsequently, the mice were placed on a heating stage that is maintained at 37 °C by running warming water through the stage using a warm water pump. Eye ointment is dabbed on the eyes and the nose is inserted in the nosepiece of the anesthesia device (1.5% isoflurane, 0.6% oxygen) during the experiment. Mice knee is clamped using a knee clamp fixed on the stage and the leg will be fixed onto the footplate that is connected to a servomotor (1300 A: 3-in-1 Whole Animal System—Mouse; Aurora Scientific). Two needle electrodes are then inserted into the tibialis anterior (TA) muscle close to the knee. The best position of muscle contraction will be determined by adjusting the distance between the footplate and the knee by stimulation with a single electrical stimulation (25 mA, 0.2 ms pulse width; conditions optimized previously). When the muscle force is no longer increasing, the position is the best position for muscle contraction. Finally, one or all of the following forces will be measured. Twitch force was measured by stimulating muscle with a single electrical stimulation (0.2 ms pulse width), repeating 3 times with an interval of 30 s. The duration of the experiment is about 2 min. The tetanic force was measured by stimulating muscle with 500 ms duration, and 0.2 ms pulse width at a series of frequencies from 10 to 200 Hz (10, 20, 35, 50, 65, 80, 100, 150, 200 Hz) with an interval of 2 min. The duration of the experiment is 20 min maximum. At the end of the experiments, mice will be placed back in the cages with a warming pad and monitored until they wake up and behave normally. Data were analyzed using 611 A Dynamic Muscle Analysis (DMA) software.

### Mouse myoblast isolation and culture

were isolated from wild-type and LAKI-Nanog (LmnaG609G/G609G, Col1a1 tetO-Nanog/+; ROSA26rtTA/rtTA) mice as described previously^61^. Isolated myoblast cells were then plated onto matrigel-coated (0.1 mg/ml) (CORNING, Corning, NY) dishes. High glucose Dulbecco’s Modified Eagle Medium (DMEM) was used for myoblast culture (Waltham, MA), 20% fetal bovine serum (FBS, Atlanta Biologicals, Flowery Branch, GA), 10% horse serum (Grand Island, NY), 0.5% chicken embryo extract (CEE, Accurate Chemical and Scientific, Westbury, NY), 2.5 ng/ml bFGF (ORF Genetics, Iceland), 10 μg/ml gentamycin (Waltham, MA), and 1% Antibiotic-Antimycotic (Grand Island, NY), and 2.5 μg/ml plasmocin prophylactic (Invivogen, San Diego, CA).

For differentiation, the cells were allowed to reach 100% confluence (in 2–3 days) and then the medium was switched to differentiation medium that was composed of DMEM with high glucose, 5% horse serum (HS), and 1% AA to promote the formation of multinucleated myotubes.

### Human myoblast cell culture

Human myoblasts from three donors (25-year female, 68-year male, and 75-year female) were purchased from Cook MyoSite (Pittsburgh, PA). The cells were seeded in Matrigel (0.1 mg/ml) (CORNING, Corning, NY) coated flasks and expanded in skeletal muscle cell Growth Medium (GM) as described previously^28^.

### Cloning and lentivirus transduction

For controlled expression of NANOG, a tetracycline-regulatable system which expresses NANOG upon the addition of a tetracycline analog, doxycycline was employed. To generate this vector, hNANOG-IRES-PURO was extracted from pSIN-hNANOG-IRES-PURO (Addgene, Cambridge, MA) and inserted into the pNL-EGFP/TREPittdU3 (Addgene) to create TRE-hNANOG-IRES-PURO. Next, the Ubiquitin C constitutive promoter and the reverse tetracycline transactivator (UbC-rtTA) was extracted from FUdeltaGW-rtTA (Addgene) and inserted into TRE-hNANOG-IRES-PURO vector using NheI and BstBI restriction sites (between hNANOG and IRES). The final plasmid is a lentiviral vector which expresses rtTA and the selection marker puromycin-*N*-acetyl-transferase under Ubiquitin C constitutive promoter and NANOG expression under a tetracycline-regulatable element (TRE) promoter (Fig. S1b). All restriction enzymes were ordered from Thermo Fisher Scientific (Waltham, MA). The p16 promoter was cloned into the LVDP vector that was previously developed in our lab^62^. For cloning the p16 promoter, we used the primers in Supplementary Table 2^63^.

The vector pCS_SMAR8_pA1_DRE2_hPGK_cHS4_Tactb_SPA_ZsG_MCS was used for promoter cloning via the ClaI/AgeI restriction sites. The resulting LVDP vector encodes for ZsGreen under the p16 promoter and for DsRed under a constitutive promoter (hPGK) (Fig S1a).

Gene knockdown was performed using the lentiviral vector EZ-Tet-pLKO-Puro from addgene (Plasmid# 85966) as described in ref. ^64^. This vector was used for shRNA cloning via the NheI/EcoRI restriction sites. shRNA targeting sequences were chosen from the BLOCK-iT™ RNAi Designer database (Invitrogen Block-iT RNAi Designer (thermofisher.com)) for human MAT2A or mouse Mat2a and they are in Supplementary Table 3.

Lentiviral plasmids were used to produce the virus as detailed previously^62^. Human and mouse myoblast cells were transduced with lentiviruses in the presence of 8 μg/ml polybrene (Sigma-Aldrich, St. Louis, MO) in GM for 6 h. Two days after transduction, the cells were selected with puromycin at 1 μg/ml for 5 days. NANOG or shRNA expression was induced by the addition of doxycycline at 1 μg/ml.

### RNA-seq and pathway analysis

RNA-sequencing (RNA-Seq) was performed with the Illumina platform to assess the global gene expression profile upon NANOG expression as previously described by ref. ^26^. Genes that were differentially expressed by >1.5-fold and with an adjusted value of *P* < 0.05 were analyzed. Differential gene expression analysis was performed using DESeq2, a variance analysis package developed to infer the statistically significant difference in RNA-seq data, and the biological hypothesis was tested using a generalized linear model implemented in DESeq2 by constructing corresponding contrast, where multiple testing correction was applied. Pathway analysis was run against MSigDB, a collection of annotated and curated gene set repositories offered by the developer of GSEA (Broad Institute of MIT and Harvard). This particular run used C2 of version 6.1 collection, containing 1329 gene sets from various well-known and up-to-date pathway databases such as BioCarta, Kyoto Encyclopedia of Genes and Genomes (KEGG), and Reactome among others.

### Senescence-associated-β-galactosidase and periodic acid schiff (PAS) stain

SA-β-Gal (ab65351, Abcam) and PAS stain (1016460001, Millipore sigma) were performed according to the manufacturer’s instructions. Cells were photographed using the Zeiss Axio Observer Z1a microscope and a 10 × objective (Plan-APOCHROMAT). The number of SA-β-Gal-positive and total cells were counted in five randomly selected fields of view (a total of >250 cells were counted per sample).

### Seahorse assay

We used Seahorse extracellular flux (XFe96) analyzer (Agilent Technologies, Santa Clara, CA) to measure the extracellular acidification rate, which is a defined measure of glycolysis. The Human and mouse myoblast cells were seeded at a density of 3000 cells/cm^2^ and 10,000 cells/cm^2^, respectively, on matrigel-coated XFe96 seahorse culture plates for 12 h. At the time of the assay, the cells were washed and culture medium was changed to seahorse assay medium for 45 min (XF DMEM medium (part number – 103575, Agilent Technologies, Santa Clara, CA) and glycolysis, glycolytic capacity and glycolytic reserve were measured after sequential injection of 10 mM glucose, 1 µM oligomycin, and 10 mM 2-DG. All calculations were based on Agilent Seahorse XF Technology white paper document or manufacturer protocol. Data were analyzed using Seahorse Wave Desktop 2.6.

### Western blots

Myoblast cells or Tibialis Anterior (TA) muscles were lysed in buffer containing 62.5 mM Tris-HCl (pH 6.8 at 25 °C), 2% (w/v) SDS, 10% (v/v) glycerol, 0.1% (w/v) bromophenol blue, 41.67 mM dithiothreitol (DTT) (Cell Signaling, Danvers, MA), and protease inhibitor cocktail (Sigma-Aldrich, St. Louis, MO). TA muscle tissues were homogenized by bead disruption and protein was isolated using the Bullet Blender, which was kept at 4 °C in a cold room. The protein concentration was determined using the Bradford assay. Lysates were denatured by incubation at 95 °C for 5 min and proteins were loaded at 45 µg per lane and were separated in 12% acrylamide gels (Waltham, MA) by SDS-polyacrylamide gel electrophoresis based on their molecular weight. After transferring proteins to nitrocellulose membranes (Bio-Rad) using the Trans-Blot Turbo Transfer System (Bio-Rad, Hercules, California), the membranes were blocked in 2% BSA (for phosphoproteins) or 5% (w/v) non-fat dry milk (for all other proteins) in blocking buffer for 1 h at room temperature. Subsequently, membranes were incubated overnight at 4 °C with antibodies listed in Supplementary Table 5. Membranes were incubated for 1 h at room temperature with Anti-rabbit IgG HRP linked (Cell Signaling, Cat# 7074; RRID: AB_2099233) or Anti-mouse IgG HRP linked (Cell Signaling, Cat# 7076; RRID: AB_330924). Finally, the protein bands were visualized using horseradish peroxidase-conjugated secondary antibodies and a chemiluminescence kit (Cell Signalling, Danvers, MA) according to the manufacturer’s instructions. Luminescent blots were imaged using ChemiDoc™ Touch Imaging System (Bio-Rad, Hercules, CA).

### Quantitative real-time PCR

Human or mouse myoblast cells were seeded in a 150 mm Petri dish (3000 cells/cm^2^). After 2 days, total RNA was isolated with RNeasy Mini Kit (Qiagen, Valencia, CA) according to the manufacturer’s instructions. cDNA was obtained using Superscript III cDNA Synthesis Kit (Invitrogen) and was subjected to real-time PCR (1 µg per reaction) using SYBR Green Kit (Bio-Rad, Hercules, CA). Primers for real-time PCR were listed in Supplementary Table 4. Bio-Rad Software CFX Manager Ver 3.1 was used for qPCR cycle determination.

### Flow cytometry

Myoblast cells were seeded at a density of 3000 cells/cm^2^ on matrigel-coated six-well plates. The next day, FBS was removed overnight the cells were incubated with a culture medium containing 20 nM insulin together with Alexa Fluor 594 conjugated antibody against Glut4 (Clone No. IF8, Cat. No: sc-53566, 1:100 dilution, Santa Cruz Biotechnology, TX) for 30 min. Cells were detached with TrypLE express (Waltham, MA), washed with 500 µl cold PBS, centrifuged at 300 × *g* at 4 °C for 5 min, and resuspended in 200 µl of cold PBS. Live cells (10,000 cells per sample) were analyzed by flow cytometry using a BD Fortessa X20, four-laser, 14-color analyzer (Franklin Lakes, NJ). The results were analyzed using FCS Express 6 Flow software.

### Glucose uptake

Myoblasts were seeded at a density of 3–10,000 cells/cm^2^ on Matrigel-coated 96-well white bottom plates (Cat No. 3903, CORNING, Corning, NY). Next, selective inhibitors for either Akt1 (A-674563, Cat No. S2670; Selleckchem, TX) or Akt2 (CCT128930, Cat No. S2635, IC50 Selleckchem, Houston, TX) in FBS-free medium were added to the cells overnight. On the next day, cells were inoculated with a culture medium containing 20 nM insulin combined with 1 mM GA for 30 min. At that point, excess GA was washed in PBS and phosopho-GA was measured using a luminometer as per the manufacturer’s specifications using Glucose Uptake‐Glo™ Assay (Promega Corporation, Madison, WI) according to the manufacturer’s instructions.

### ATP, *s*-adenosyl methionine, *s*-adenosylhomocysteine, and ammonia measurement assays

Myoblasts were seeded on Matrigel-coated 96-well white plates (human cells at 3000 cells/cm^2^ and mouse cells at 10,000 cells/cm^2^). The next day, human myoblasts were switched to only 12.5 mM glucose-containing medium without pyruvate, glutamine, or serum for overnight. Mouse myoblasts were cultured in a serum-free medium with glucose, pyruvate, and glutamine until the point of the assay. Cells were treated with different combinations of Glucose (6.25, 12.5, and 25 mM), β-2-chloro-alanine (250 μM), etomoxir (40 μM), 2-DG (1, 5, and 10 mM), FIDAS V (1, 2, and 5 μM), or shMAT2A_1/shMAT2A_2 in complete medium overnight. For SAH and SAM measurements, ELISA Combo Kit (Cell Biolabs, San Diego, CA) was used; intracellular ATP levels were measured using a luminescent ATP detection assay kit (ab113849, Abcam, Cambridge, MA); and ammonia was measured using the ammonia Assay Kit (ab83360, Abcam, Cambridge, MA) according to the manufacturer’s instructions.

### Pyruvate kinase assay

Myoblasts were seeded at a density of 5000 cells/cm^2^ on Matrigel-coated 96-well white plates. The cells were allowed to attach overnight and the next day the cells were starved in a medium containing only 12.5 mM glucose for 6 h. Next, the cells were lysed with Pyruvate kinase assay buffer and PK activity was determined using a PK activity assay kit (ab83432, Abcam, Cambridge, MA) according to the manufacturer’s instructions. The optical density (OD570nm) was measured as a function of time using a BioTek Synergy 4 microplate plate reader at room temperature every 10 min for a total of 40 min.

### Tissue embedding and immunostaining

TA muscles were isolated and fixed in 10% formalin (Sigma-Aldrich, St. Louis, MO) for 6 h. The tissues were then washed with PBS and transferred to PBS + 0.1 M glycine (VWR) for 1 h, then sequentially transferred overnight each into PBS containing 15% and then 30% sucrose (Ward’s Science, Rochester, NY). The tissues were then immersed in an embedding medium (OCT, Sakura Finetek, Torrance, CA) and frozen in 2-Methylbutane (Sigma-Aldrich, St. Louis, MO) chilled with dry ice. Tissue sections (10-μm thick) were cut at −20 °C using a cryostat (LEICA CM1950, Buffalo Grove, IL) and placed on positively charged glass slides (Stellar Scientific, Baltimore, MD) and stored at −80 °C.

For immunostaining, tissue sections were washed three times in PBS to remove the OCT and then permeabilized with −20 °C methanol (Sigma-Aldrich, St. Louis, MO) for 10 min. The slides were immersed in R-Buffer A (Electron Microscopy Sciences, Hatfield, PA), and for antigen retrieval, the temperature was raised to 95 °C for 20 min and then samples were allowed to cool gradually. Endogenous peroxidase activity was quenched using Tyramide H_2_O_2_ solution (Alexa Fluor™ 555 Tyramide SuperBoost™ Kit, Thermo Fisher Scientific, Waltham, MA) for 30 min prior to blocking with 5% (w/v) goat serum and 5% (w/v) BSA in PBS for 1 h; Tyramide Blocking Buffer for 1 h; and mouse IgG blocking reagent (MOM, Vectors Lab, Burlingame, CA) for 1 h according to the manufacturer’s protocol. The tissues were then incubated overnight at 4 °C with primary antibodies diluted in MOM diluent. Primary antibodies were used in this study (Supplementary Table 5). The next day, samples were washed with PBS and stained with Tyramide kit goat anti-mouse secondary antibody according to the Tyramide kit protocol. Subsequently, the samples were stained with Alexa Fluor 568, 488, or 647 conjugated goat anti-rabbit or goat anti-mouse secondary antibodies for 1 h at room temperature.

The immunostaining of cells in culture, cells were fixed for 10 min in 4% paraformaldehyde followed by permeabilization with 0.1% (v/v) triton X-100/PBS for 10 min at RT and blocked with blocking buffer (5% (v/v) goat serum in 0.01% (w/v) triton X-100/PBS) at RT for 1 h. Next, samples were immunostained with antibodies listed in Supplementary Table 5. Subsequently, the cells were incubated with Alexa Fluor 594 or 488 conjugated goat anti-mouse or goat anti-rabbit antibodies (1:200 dilution in blocking buffer, Thermo Fisher Scientific, Waltham, MA) for 1 h at room temperature and counter-stained with Hoechst 33342 nuclear dye (1:1000 dilution in PBS, Thermo Fisher Scientific, Waltham, MA) for 5 min at RT.

### Statistical analysis

All data were collected in Excel (2017) and statistical significance was calculated in GraphPad Prism®8 software using the following tests: one-way or two-way ANOVA followed by Tukey post hoc test or Dunnett’s multiple comparisons test or Sidak’s multiple comparisons test. For comparing two conditions only, we used the paired Student’s *t*-test. All values were considered statistically different when *p* < 0.05. Each experiment was performed at least three times, each time with samples at least in triplicate. Statistical significance was denoted as *p < 0.05, **p < 0.01, ***p < 0.001, and ****p < 0.0001.

### Reporting summary

Further information on research design is available in the Nature Portfolio Reporting Summary linked to this article.

## Supplementary information

Supplementary Information

Description of Additional Supplementary Files

Supplementary Movie 1

Supplementary Movie 2

Supplementary Movie 3

Supplementary Movie 4

Reporting Summary

## Data Availability

The data generated in this study are provided in the Supplementary Information/Source Data file, even if the source data file has also been uploaded on Mendeley at https://data.mendeley.com/datasets/s5zgs8xh7c/5, 10.17632/s5zgs8xh7c.5. The RNA-seq data discussed in this publication are accessible through Sequence Read Archive (SRA) Series accession number PRJNA914041. Source data are provided with this paper.

## Source data

Source Data
