# Supplementary material for: Methionine adenosyltransferase2A inhibition restores metabolism to improve regenerative capacity and strength of aged skeletal muscle
Source: Nat Commun. 2023 Feb 16;14:886. doi: 10.1038/s41467-023-36483-3 (PMC9935517; doi:10.1038/s41467-023-36483-3)
Supplement: Supplementary file 1 — Supplementary Information [file 41467_2023_36483_MOESM1_ESM.pdf]

Supplementary Figure 1

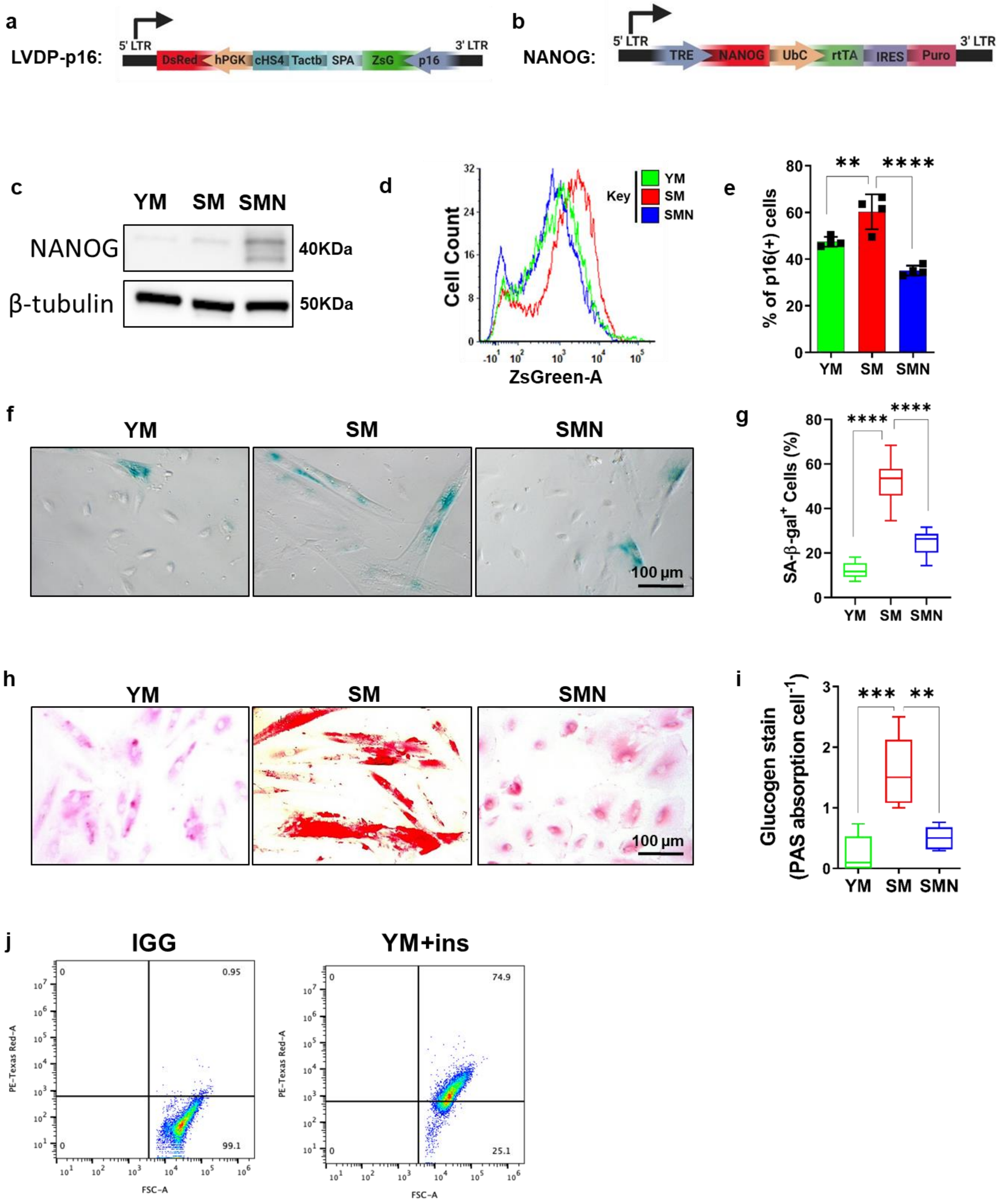

Supplementary Figure 1. a Schematic of the vector containing two expression cassettes in trans of the lentiviral backbone: one encodes for ZsGreen under the p16 promoter; the other encodes for DsRed under the constitutive hPGK promoter. **b** Schematic of the lentivirus encoding for NANOG under the tetracycline-regulatable promoter (TRE); the reverse tetracycline trans-activator, rtTA is expressed constitutively under the ubiquitin (UbC) promoter, which also drives puromycin phosphotransferase downstream of IRES. **c** Western blots for NANOG in human myoblast cells. β-tubulin served as loading control. **d,e** Flow cytometry for p16 and percentage p16+ cells transduced with LVDP-p16 vector**.** Unstained cell which incubated with flurochrome control were used to gate immunopositive shift. **f,g** Staining for SA-β-Gal in YM, SM and SMN cells and quantification of the percentage of SA-β-Gal-positive cells (n = 250 cells) (scale bar = 100 μm). Statistical significance using one-way ANOVA with Tukey's multiple comparisons test (*** p = 0.009, and **** p < 0.0001). **h,i** PAS stain for glycogen accumulation YM, SM, and SMN and quantification for Schiff absorbance per cell (n=250 cells) (scale bar=100μm). Statistical significance using one-way ANOVA with Tukey's multiple comparisons test (** p < 0.01, and *** p < 0.001). **j** For flow cytometry gating, the corresponding IgG conjugated antibody was used to establish proper gating of positive/negative cell populations. Young myoblast: YM, Senescent myoblast: SM, and Senescent NANOG myoblast: SMN. YM: green bars or green lines, SM: red bars or red lines, and SMN: blue bars or blue lines. n=3 human donors/group. Data in bar graphs are presented as mean ± SD. For the boxplots, the top and bottom lines of each box represent the 75th and 25th percentiles of the samples, respectively. The line inside each box represents the median of the samples. The upper and lower lines above and below the boxes are the whiskers.

Supplementary Figure 2

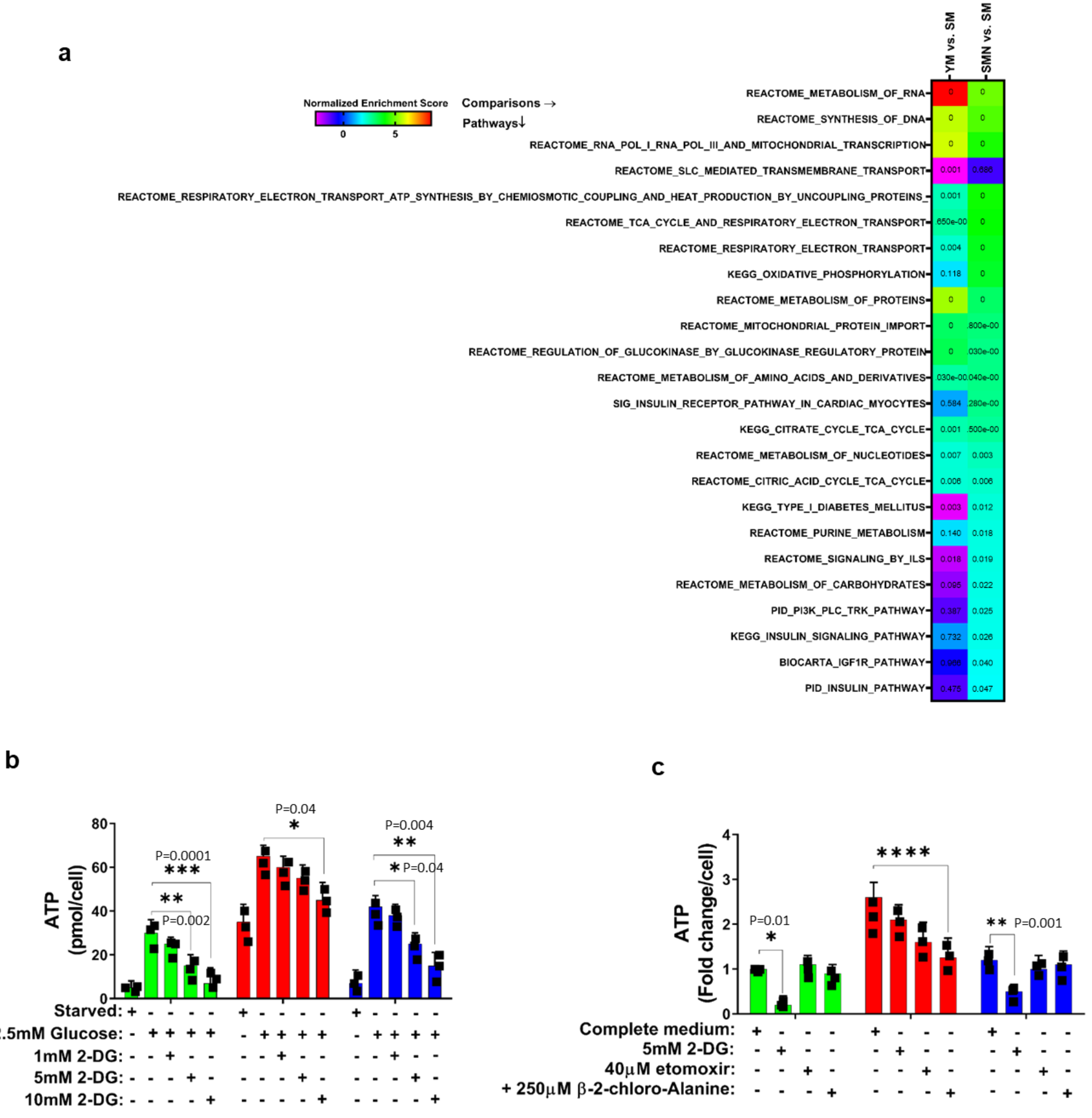

Supplementary Figure 2. a A heat map of representative metabolic pathways that are significantly enriched after 12 days of NANOG expression (FDR is written inside the cells, and when FDR<0.001 is depicted as 0). **b** The levels of ATP level measured in the presence of the indicated concentrations of 2-DG (hexokinase inhibitor, IC_{50}=5mM) in 12.5mM glucose containing medium. **c** ATP levels of YM, SM, and SMN in the presence of 2-DG, Etomoxir or β-2-chloro-alanine inhibitors. Statistical significance using two-way ANOVA with Tukey's multiple comparisons test. YM: green bars, SM: red bars, and SMN: blue bars. n=3 human donors/group. Data in bar graphs are presented as mean ± SD.

Supplementary Figure 3

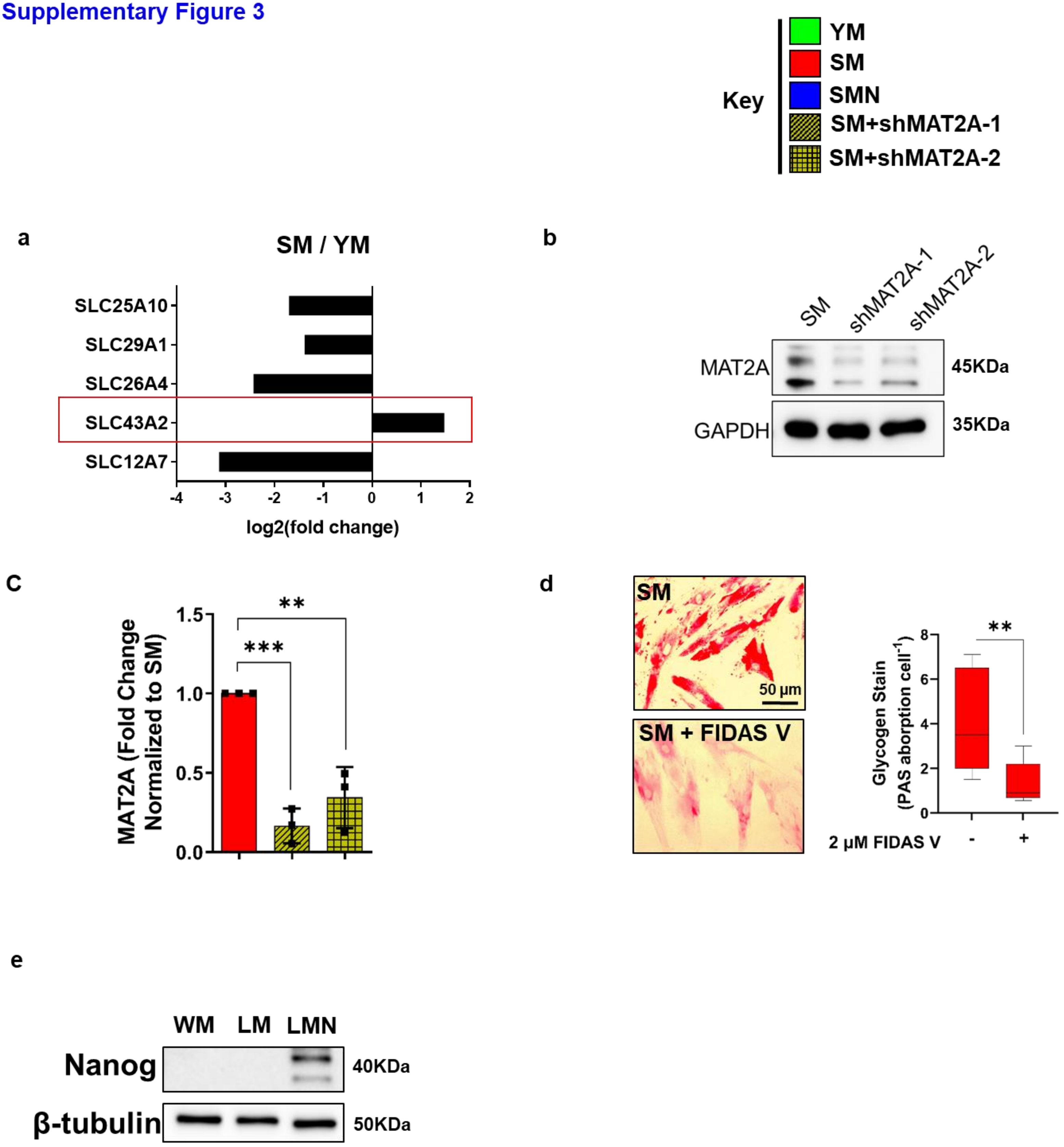

Supplementary Figure 3. a Changes in gene expression of representative solute transporters (SLC) in SM/YM as measured by RNA-seq. **b** Western blot and band quantification of MAT2A knockdown in SM cells using two distinct shRNAs. GAPDH served as loading control. Statistical significance using one-way ANOVA with Tukey's multiple comparisons test. (** $p = 0.001$, and *** $p = 0.005$). **d** PAS stain for glycogen accumulation SM and SM+FIDAS V and quantification for Schiff absorbance per cell (n=250 cells) (scale bar=50μm). (**$p < 0.001$ using unpaired t-test). n=3 human donors/group. **e** Western blots for NANOG in mouse myoblast cells. β-tubulin served as loading control. n=3 human donors/group. SM: red bars, and SM+shMAT2A-1,2: yellow bars. Data in bar graphs are presented as mean ± SD. For the boxplots, the top and bottom lines of each box represent the 75th and 25th percentiles of the samples, respectively. The line inside each box represents the median of the samples. The upper and lower lines above and below the boxes are the whiskers.

Supplementary Figure 4

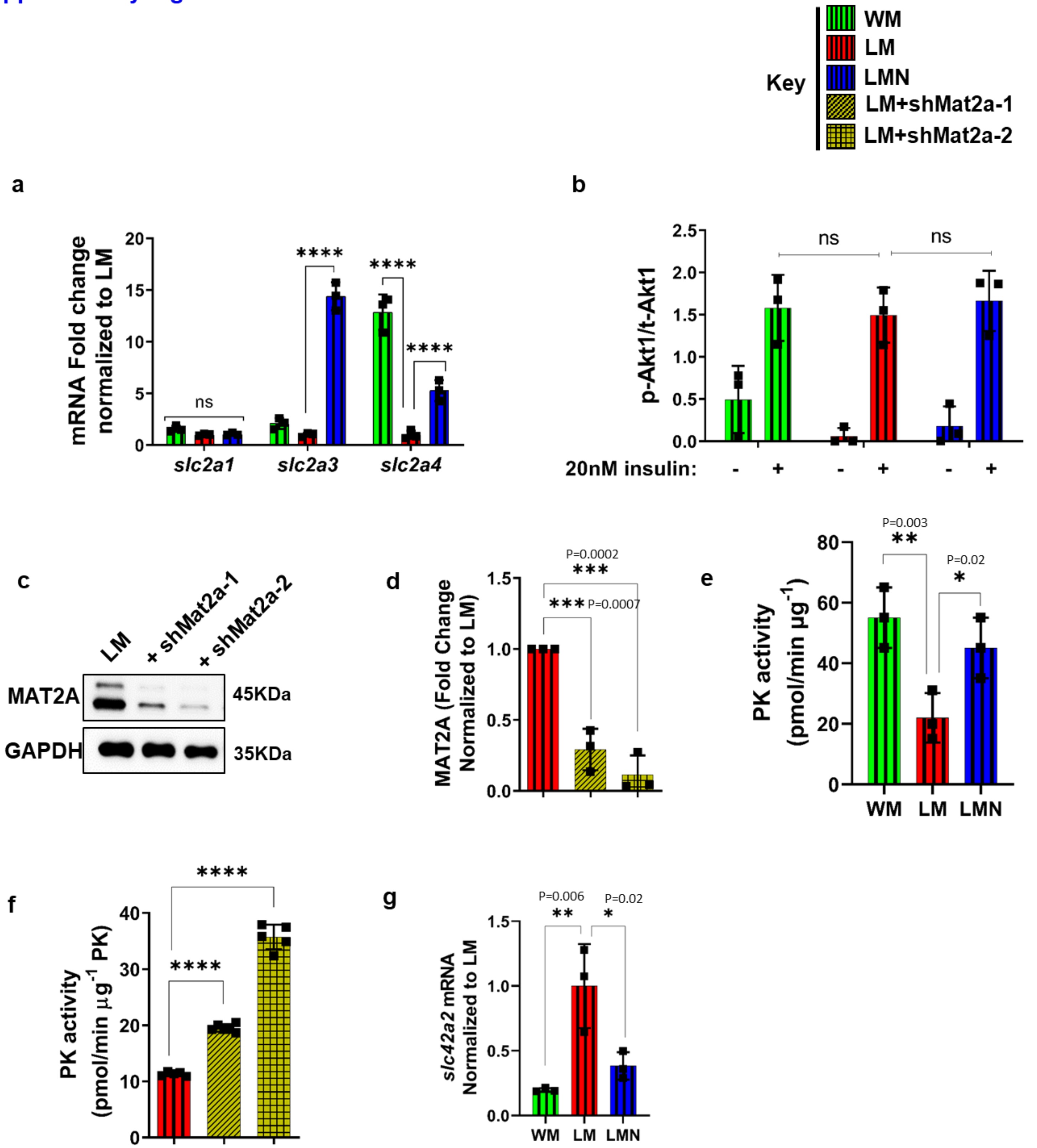

Supplementary Figure 4. a Effect of NANOG on mouse *slc2a1*, *slc2a3* and *slc2a4* transcripts. Statistical significance using two-way ANOVA with Tukey's multiple comparisons test. (**** $p < 0.0001$, and ns represents $p > 0.6$). **b** Band quantification of western blot for the effect of insulin on mouse Akt1 and p-Akt1; GAPDH served as loading control. (ns represents $p > 0.3$) statistical significance using two-way ANOVA with Sidak's multiple comparisons test. **c,d** Western blot and band quantification of MAT2A knockdown in LM cells using two distinct shRNAs. GAPDH served as loading control. **e** Effect of NANOG on mouse myoblast PK activity. **f** Effect of MAT2A inhibition on LM PK activity. **g** Effect of NANOG on mouse *slc42a2* transcripts. Statistical significance using one-way ANOVA with Tukey's multiple comparisons test. (**** $p < 0.0001$). WM: green bars, LM: red bars, LMN: blue bars, and LM+shMat2a-1,2: yellow bars. n=5 mice donors/group. Data in bar graphs are presented as mean ± SD.

Supplementary Figure 5

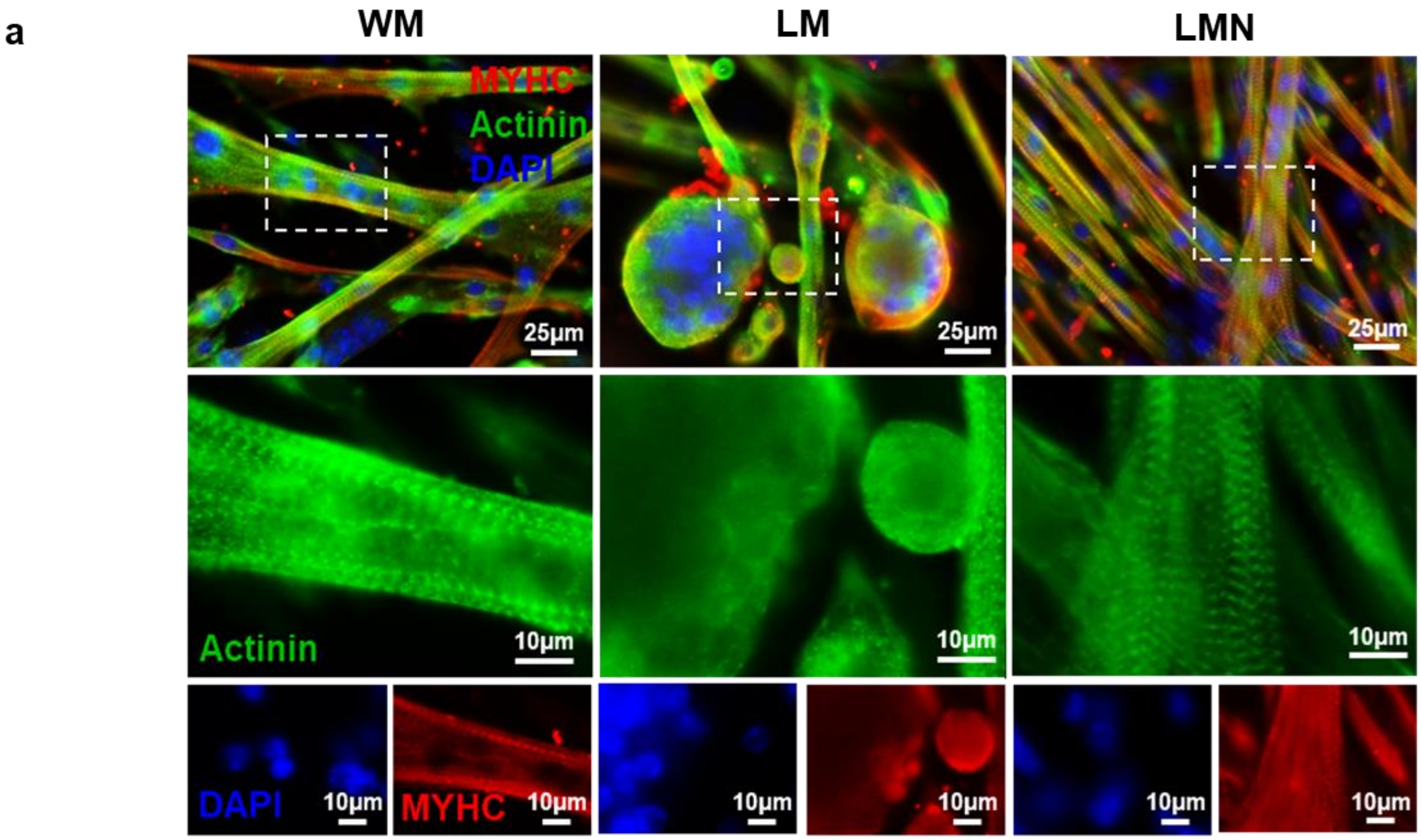

Supplementary Figure 5. a Immunostaining for myosin heavy chain (MyHC) (red) and actinin (green) showing skeletal muscle differentiation (scale bar= 25μm). **b** Videos showing contraction of differentiated mouse myofibers. n=5 mice donors/group.

Supplementary Figure 6

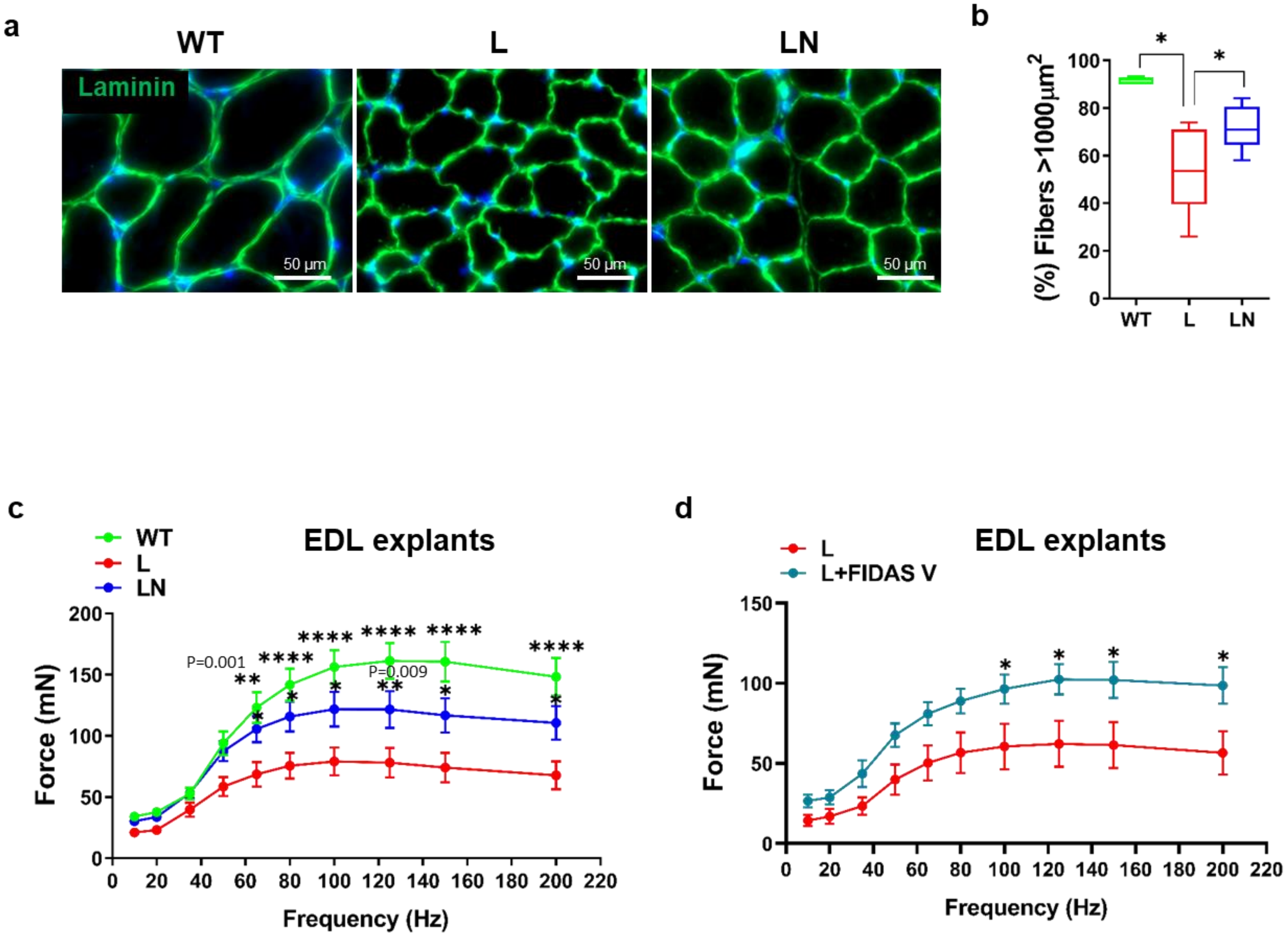

Supplementary Figure 6. a,b Immunostaining of laminin and quantification of the percentage of fibers with area > 1,000 μm^2 (scale bar= 50µm). n=6 mice/group. Statistical significance using one-way ANOVA with Tukey's multiple comparisons test. (* p = 0.01). **c** Effect of NANOG on isometric contraction performed *ex vivo* on EDL harvested from WT, L, and LN mice. n=6 mice/group. **d** Effect of FIDAS V on isometric contraction performed *ex vivo* on EDL harvested from LAKI animals. L: LAKI control; L + FIDAS V: muscles from LAKI mice that received FIDAS V. n=4 mice/group. Statistical significance using two-way ANOVA with Tukey's multiple comparisons test. (* p = 0.01, **** $p < 0.0001$). WT are green boxes or green lines, L are red boxes or red lines, LN are blue boxes or blue lines, and L+FIDAS V are light blue bars or light blue lines. n=4-6 animals per cohort. Data in lines are presented as mean ± SEM. For the boxplots, the top and bottom lines of each box represent the 75th and 25th percentiles of the samples, respectively. The line inside each box represents the median of the samples. The upper and lower lines above and below the boxes are the whiskers.

Supplementary Table 1

**Target gene**	**Sequence 5'→ 3'**
**Nanog primers**	CCCTCCATGTGTGACCAAGG-common
	GCACAGCATTGCGGACATGC-wt
	GCAGAAGCGCGGCCGTCTGG-tg
**rtTA primers**	AAAGTCGCTCTGAGTTGTTAT-common
	GCGAAGAGTTTGTCCTCAACC-mut F
	GGAGCGGGAGAAATGGATATG-mut R
**lmna primers**	GGTTCCCACTGCAGCGGCTC- mm-lmna forward exon 1
	GGACCCCACTCCCTTGGGCT-mm-lmna reverse (intron)

Supplementary Table 2

Target gene	Sequence 5'→ 3'
**p16-F**	CGGATCGATTACAGGTGATTTCGATTTCTCGGTGGGG
**p16-R**	ATTACCGGTGGCACCAGCCGGAAGCAGCC

Supplementary Table 3

Target gene	Sequence 5'→ 3'
**MAT2A-1**	GCAACAGTCACCAGATATTGC
**MAT2A-2**	GGGATGCCCTAAAGGAGAAAG
**Mat2a-1**	GGTCACCCAGATAAGATTTGT
**Mat2a-2**	GCCATAAAGCACATTGGATAT

Supplementary Table 4

Target gene	Forward 5'→ 3'	Reverse 5'→ 3'
**MAT2A-human**	ATGAACGGACAGCTCAACGG	CCAGCAAGAAGGATCATTCCAG
**RPL-32-human**	GCCCAAGATCGTCAAAAAGAGA	TCCGCCAGTTACGCTTAATTT
**slc2a1-mouse**	GCAGTTCGGCTATAACACTGG	GCGGTGGTTCCATGTTTGATTG
**slc2a3-mouse**	ATGGGGACAACGAAGGTGAC	CAGGTGCATTGATGACTCCAG
**slc2a4-mouse**	ACACTGGTCCTAGCTGTATTCT	CCAGCCACGTTGCATTGTA
**slc42a2-mouse**	TGCTATCTTTGTCCGGTACAAC	AGCTTGGGTAGCGAAAGTAAAAC
**Rp-11β-mouse**	GGTCAGAAGGGAACTTGTGGTAT	GCATCATTAAATGGAGTAGCGTC

Supplementary Table 5

Antibody(Clone#)	Catalog#	Dilution	Company
**RHCG(5A4)**	WH0051458M6	WB: 1:1000	Millipore
**Akt1(9Q7)**	AHO1112	WB: 1:1000	Invitrogen
**pAkt1- Ser473(14-6)**	44-621G	WB: 1:1000	Invitrogen
**Akt2 (4H7)**	MA1-034	WB: 1:1000	Invitrogen
**pAkt2-Ser474**	PA5-104870	WB: 1:1000	Invitrogen
**InsR (CT-3)**	MA5-13783	WB: 1:250	Invitrogen
**MAT2A**	NB110-94158	WB: 1:1000, IF: 1:200	Novus Biological
**GAPDH**	5174	WB: 1:10000	Cell signaling
**Pax7**	AB-528428	IF:1:10	Developmental Studies Hybridoma Bank
**Laminin**	L9393	IF: 1:200	Sigma-Aldrich
**Sarcomeric Alpha Actinin**	ab68167	IF: 1:200	Abcam
**MyHC(clone A4.1025)**	05-716	IF: 1:200	Millipore
**eMyHC**	Sc-53091	IF:1:100	Santa Cruz Biotechnology
**NANOG**	Ab109250	WB:1:500, IF: 1:250	Abcam
