# Supplementary material for: Methionine adenosyltransferase2A inhibition restores metabolism to improve regenerative capacity and strength of aged skeletal muscle
Source: Nat Commun. 2023 Feb 16;14:886. doi: 10.1038/s41467-023-36483-3 (PMC9935517; doi:10.1038/s41467-023-36483-3)
Supplement: Supplementary file 2 — Description of Additional Supplementary Files [file 41467_2023_36483_MOESM2_ESM.pdf]

Description of Additional Supplementary Files

Supplementary Movie 1: Spontaneous contraction in myotubes derived from wild type mouse myoblast (WM) cells in vitro.

Supplementary Movie 2: Spontaneous contraction in myotubes derived from LAKI mouse myoblast (LM) cells in vitro.

Supplementary Movie 3: Spontaneous contraction in myotubes derived from LAKI-NANOG mouse myoblast (LMN) cells in vitro.

Supplementary Movie 4: Spontaneous contraction in myotubes derived from LAKI mouse myoblast with shMat2a (LM+shMat2a) cells in vitro.
