# Supplementary material for: Methionine adenosyltransferase2A inhibition restores metabolism to improve regenerative capacity and strength of aged skeletal muscle
Source: Nat Commun. 2023 Feb 16;14:886. doi: 10.1038/s41467-023-36483-3 (PMC9935517; doi:10.1038/s41467-023-36483-3)
Supplement: Supplementary file 7 — Reporting Summary [file 41467_2023_36483_MOESM7_ESM.pdf]

nature research

Corresponding author(s): Stelios Andreadis

Last updated by author(s): 12/20/2022

Reporting Summary

Nature Research wishes to improve the reproducibility of the work that we publish. This form provides structure for consistency and transparency in reporting. For further information on Nature Research policies, see Authors & Referees and the Editorial Policy Checklist .

Statistics

For all statistical analyses, confirm that the following items are present in the figure legend, table legend, main text, or Methods section.

n/a	Confirmed	
☐	☒	The exact sample size (n) for each experimental group/condition, given as a discrete number and unit of measurement
☐	☒	A statement on whether measurements were taken from distinct samples or whether the same sample was measured repeatedly
☐	☒	The statistical test(s) used AND whether they are one- or two-sided<br>*Only common tests should be described solely by name; describe more complex techniques in the Methods section.*
☒	☐	A description of all covariates tested
☐	☒	A description of any assumptions or corrections, such as tests of normality and adjustment for multiple comparisons
☐	☒	A full description of the statistical parameters including central tendency (e.g. means) or other basic estimates (e.g. regression coefficient) AND variation (e.g. standard deviation) or associated estimates of uncertainty (e.g. confidence intervals)
☐	☒	For null hypothesis testing, the test statistic (e.g. F, t, r) with confidence intervals, effect sizes, degrees of freedom and P value noted<br>*Give P values as exact values whenever suitable.*
☒	☐	For Bayesian analysis, information on the choice of priors and Markov chain Monte Carlo settings
☒	☐	For hierarchical and complex designs, identification of the appropriate level for tests and full reporting of outcomes
☒	☐	Estimates of effect sizes (e.g. Cohen's d, Pearson's r), indicating how they were calculated

Our web collection on statistics for biologists contains articles on many of the points above.

Software and code

Policy information about availability of computer code

Data collection	no software were used for Data collection
Data analysis	ImageJ 1.52p, GraphPad Prism®8, Excel 2017, FCS Express 6 Flow, 611A Dynamic Muscle Analysis (DMA) software, Spike 2 software (Windows, version 6, CED Products), BioRad Software CFX Manager Ver 3.1 was used for qPCR cycle determination, Seahorse Wave Desktop 2.6 (Agilent technologies). The optical density was measured as a function of time using a BioTek Synergy 4 microplate plate reader.<br>Differential gene expression analysis was performed using DESeq2 , a variance analysis package developed to infer the statistically significant difference in RNA-seq data, and biological hypothesis was tested using generalized linear model implemented in DESeq2 by constructing corresponding contrast, where multiple testing correction was applied.<br>Pathway analysis was run against MSigDB, a collection of annotated and curated gene set repositories offered by the developer of GSEA (Broad Institute of MIT and Harvard). This particular run used C2 of version 6.1 collection, containing 1329 gene sets from various well-known and up-to-date pathway databases such as BioCarta, Kyoto Encyclopedia of Genes and Genomes (KEGG), and Reactome among others.

For manuscripts utilizing custom algorithms or software that are central to the research but not yet described in published literature, software must be made available to editors/reviewers. We strongly encourage code deposition in a community repository (e.g. GitHub). See the Nature Research guidelines for submitting code & software for further information.

Data

Policy information about availability of data

All manuscripts must include a data availability statement. This statement should provide the following information, where applicable:
- Accession codes, unique identifiers, or web links for publicly available datasets
- A list of figures that have associated raw data
- A description of any restrictions on data availability

The data that support the findings of this study are openly available in "Mendeley Data" at https://data.mendeley.com/datasets/s5zgs8xh7c/5. The RNA-seq data discussed in this publication are accessible through Sequence Read Archive (SRA) Series accession number PRJNA914041.

Field-specific reporting

Please select the one below that is the best fit for your research. If you are not sure, read the appropriate sections before making your selection.

☒ Life sciences ☐ Behavioural & social sciences ☐ Ecological, evolutionary & environmental sciences

For a reference copy of the document with all sections, see nature.com/documents/nr-reporting-summary-flat.pdf

Life sciences study design

All studies must disclose on these points even when the disclosure is negative.

Sample size	by calculation p value, All values were considered statistically different when p< 0.05.
Data exclusions	No exclusions
Replication	Each donor was performed in triplicates. Repeats yielded similar results.
Randomization	treatments were assigned in random with a group of mice of same age and strain.
Blinding	Blinding was not possible. Tissue processing and cell culture were performed by designated individuals and experimental results were performed and re-verified by different groups of scientists.

Reporting for specific materials, systems and methods

We require information from authors about some types of materials, experimental systems and methods used in many studies. Here, indicate whether each material, system or method listed is relevant to your study. If you are not sure if a list item applies to your research, read the appropriate section before selecting a response.

Materials & experimental systems

n/a	Involved in the study	
☐	☒	Antibodies
☒	☐	Eukaryotic cell lines
☒	☐	Palaeontology
☐	☒	Animals and other organisms
☒	☐	Human research participants
☒	☐	Clinical data

Methods

n/a	Involved in the study	
☒	☐	ChIP-seq
☐	☒	Flow cytometry
☒	☐	MRI-based neuroimaging

Antibodies

Antibodies used

RHCG(5A4) WH0051458M6 WB: 1:1000 Millipore
Akt1(9Q7) AHO1112 WB: 1:1000 Invitrogen
pAkt1- Ser473(14-6) 44-621G WB: 1:1000 Invitrogen
Akt2 (4H7) MA1-034 WB: 1:1000 Invitrogen
pAkt2-Ser474 PA5-104870 WB: 1:1000 Invitrogen
InsR (CT-3) MA5-13783 WB: 1:250 Invitrogen
MAT2A NB110-94158 WB: 1:1000, IF: 1:200 Novus Biological
GAPDH 5174 WB: 1:10000 Cell signaling
Myosin Heavy Chain (clone A4.1025)05-716 IF: 1:500 Millipore
Sarcomeric Alpha Actinin ab68167 IF: 1:200 Abcam
Alexa Fluor 594 conjugated antibody against Glut4 (Clone No. IF8) Cat. No: sc-53566, 1:100 dilution, Santa Cruz Biotechnology

Pax7 AB-528428 IF:1:10 Developmental Studies Hybridoma Bank
Laminin L9393 IF: 1:200 Sigma-Aldrich
eMyHC Sc-53091 IF:1:100 Santa Cruz Biotechnology
NANOG Ab109250 WB:1:500, IF: 1:250 Abcam
Anti-rabbit IgG HRP linked (Cell Signaling, Cat# 7074)
Anti-mouse IgG HRP linked (Cell Signaling, Cat# 7076)

Validation	All antibodies were validated using myoblast cells in addition to the validation performed by the companies. Specificity of each antibody to each antigen was confirmed by negative control (without defined antigen).

Animals and other organisms

Policy information about studies involving animals; ARRIVE guidelines recommended for reporting animal research

Laboratory animals	Mice, C57BL/6, Female and Male, 3-4 month, The mice were housed with a 12hr light/dark cycle between 6:00 and 18:00 in a temperature-controlled room (22 oC) with free access to food and water. Humidity is to be between 30-70%.
Wild animals	No wild animals were used in the study.
Field-collected samples	No field collected samples were used in the study.
Ethics oversight	All animal research protocols were approved by Institutional Animal Care and Use Committee (IACUC) of the University at Buffalo in compliance with the Animal Welfare Act, Public Health Service Policy on humane care and use of laboratory animals and other federal statutes and regulations relating to animals and experiments involving animals.

Note that full information on the approval of the study protocol must also be provided in the manuscript.

Flow Cytometry

Plots

Confirm that:

- [x] The axis labels state the marker and fluorochrome used (e.g. CD4-FITC).
- [x] The axis scales are clearly visible. Include numbers along axes only for bottom left plot of group (a 'group' is an analysis of identical markers).
- [x] All plots are contour plots with outliers or pseudocolor plots.
- [x] A numerical value for number of cells or percentage (with statistics) is provided.

Methodology

Sample preparation	Myoblast cells were seeded at a density of 3,000 cell/cm2 on Matrigel coated 6-well plates. The next day, myoblasts were weaned from serum by overnight withdrawal of FBS. The cells were inoculated with culture medium containing 20nM insulin together with antibody against Glut4 (Clone No. IF8, Cat. No: sc-53566, 1:100 dilution, Santa Cruz Biotechnology, TX) conjugated to Alexa Fluor 594 for 30 mins. Cells were detached with TrypLE express (Invitrogen) and suspended in 500 µl PBS in 1.5 ml microcentrifuge tube. Next, the samples were washed with cold PBS and the cells were centrifuged at 300 × g at 4°C for 5 minutes. Following washing, the supernatant was discarded and resuspended in 200 µl of cold PBS. After staining, live cells were analyzed by flow cytometry analysis of 10,000 cells per sample using a BD Fortessa X20, four-laser, 14-color analyzer (Biosciences, CA). The results were analyzed using FCS Express 6 Flow.
Instrument	BD Fortessa X20, four-laser, 14-color analyzer (Biosciences, CA)
Software	FCS Express 6 Flow
Cell population abundance	Analyzed 10000 events per sample.
Gating strategy	Gating for positive or negative populations was determined via use of conjugated IGG controls as specified.

- [x] Tick this box to confirm that a figure exemplifying the gating strategy is provided in the Supplementary Information.
